# Capillin, a Polyacetylene from *Argyranthemum frutescens*, a Promising Hit Compound for Antikinetoplastid Drug Discovery

**DOI:** 10.3390/pathogens15101047

**Published:** 2026-10-02

**Authors:** Suhib Muhsen Hasan Al-Dalaeen, Carlos J. Bethencourt-Estrella, Ines Sifaoui, Atteneri López-Arencibia, María González-Linares, Isabel L. Bazzocchi, Jacob Lorenzo-Morales, Ignacio A. Jiménez, José E. Piñero

**Affiliations:** 1 Instituto Universitario de Enfermedades Tropicales y Salud Pública de Canarias, Universidad de La Laguna, 38296 San Cristóbal de La Laguna, Spain; alu0101616759@ull.edu.es (S.M.H.A.-D.); isifaoui@ull.edu.es (I.S.); atlopez@ull.edu.es (A.L.-A.); jmlorenz@ull.edu.es (J.L.-M.); jpinero@ull.edu.es (J.E.P.); 2 Departamento de Obstetricia y Ginecología, Pediatría, Medicina Preventiva y Salud Pública, Toxicología, Medicina Legal y Forense y Parasitología, Universidad de La Laguna, C/Sta. María Soledad s/n, 38200 San Cristóbal de La Laguna, Spain; 3 Consorcio Centro de Investigación Biomédica en Red, Área de Enfermedades Infecciosas (CIBERINFEC) Instituto de Salud Carlos III, Av. Monforte de Lemos 3–5, Pabellón 11, 28029 Madrid, Spain; 4 Instituto Universitario de Bio-Orgánica Antonio González, Departamento de Química Orgánica, Universidad de La Laguna, Avenida Astrofísico Francisco Sánchez 2, 38206 San Cristóbal de La Laguna, Spain; alu0101339009@ull.edu.es (M.G.-L.); ilopez@ull.edu.es (I.L.B.)

**Keywords:** *Argyranthemum frutescens*, bioassay-guided fractionation, polyacetylenes, antikinetoplastid compounds, mechanisms of action

## Abstract

Chagas disease and leishmaniasis, caused by the protozoan *Trypanosoma cruzi* and different *Leishmania* species, respectively, remain public health problems in developing regions due to high prevalence and the limited efficacy and safety of current treatments. Plant-derived natural products are emerging as a promising source of safer antikinetoplastid agents. The present work explores *Argyranthemum frutescens* as a source of antiparasitic compounds to identify new therapeutic candidates. To address this aim, a bioassay-guided fractionation of the ethanolic root extract from cultivated *A. frutescens* was carried out against kinetoplastids, including *T. cruzi*, *L. amazonensis*, and *L. donovani*. Polyacetylenes with leishmanicidal and trypanocidal activity were identified using this approach. Among them, compound **1** showed greater activity than the reference drug miltefosine against *L. amazonensis* and *T. cruzi*. Compound **1** showed activity against the amastigote stages of both parasites, surpassing the activity of the leishmanicidal reference drug. Notably, compound **1** showed a good selectivity index against the mammalian cell line. The findings indicated that the nature of the chemical group present at the propargyl–benzyl position plays a role in compound bioactivity. Subsequent assays revealed that this compound induces programmed cell death-related processes in *T. cruzi* and *L. amazonensis*, including ATP depletion, chromatin condensation, plasma membrane damage, accumulation of reactive oxygen species (ROS), and alterations in mitochondrial membrane potential (ΔΨm). Collectively, these findings highlight the potential of polyacetylenes as promising antikinetoplastid lead compounds and identify *A. frutescens* as a valuable natural source for the development of therapeutic agents against Chagas disease and leishmaniasis.

## 1. Introduction

Parasitic diseases represent one of the most significant public health challenges in developing countries. Many of these diseases are neglected, owing to their high prevalence in low-income settings and their limited global visibility. Predominantly, these countries are found in tropical regions. Furthermore, the climates of these regions have been demonstrated to play a pivotal role in the proliferation of parasitic infections. The presence of high humidity and temperatures has been identified as a crucial factor that promotes the growth of both vectors and protozoan parasites [1]. Among the most significant Neglected Tropical Diseases (NTDs) identified by the World Health Organization (WHO) are leishmaniasis and Chagas disease.

Chagas disease, caused by the parasite *Trypanosoma cruzi*, is transmitted to humans through the bites and faeces of triatomine insects, commonly known as “kissing bugs” [2,3]. In fact, the majority of Chagas disease outbreaks in humans are associated with contaminated food. Additionally, the parasite can be transmitted through mother-to-child transmission or organ transplantation. Most cases are found in Latin America, where it is estimated that 6–7 million people are infected. However, there has been an increase in the number of cases being detected in other countries due to travel and migration [4]. If left untreated, American trypanosomiasis can become a life-threatening condition, leading to severe cardiac, gastrointestinal, and neurological complications.

Furthermore, leishmaniasis is one of the most widespread neglected tropical diseases (NTDs) in the world, with 88 endemic countries, more than 11 million infected individuals and 350 million at risk of infection, according to recent reports [5,6]. Leishmaniasis is transmitted through the bite of infected sandflies and is caused by parasites of the genus *Leishmania*. Common symptoms of leishmaniasis include fever, anemia, weight loss, hepatomegaly, splenomegaly and weakened immunity [7].

Current treatments for these infections rely on the administration of benznidazole and nifurtimox for Chagas disease and miltefosine, pentavalent antimonials, amphotericin B, or paromomycin for leishmaniasis [8]. Despite the availability of therapeutic options, these drugs have shown significant limitations over the past 50 years, including toxicity, the emergence of resistance, and prolonged treatments [9,10].

Natural products have historically played a crucial role in the treatment of tropical parasitic diseases. Between 1981 and 2019, a total of 20 antiparasitic chemical entities derived from natural products and/or their synthetic derivatives were reported [11]. Furthermore, plant extracts have been used for centuries by traditional cultures as primary, and often the only available, treatments for a wide range of infectious diseases [12]. Recently, there has been a notable increase in research focusing on antikinetoplastid drugs, particularly natural products from medicinal plants [13].

The *Asteraceae* family, historically known as Compositae, is the largest flowering plant family, widely distributed across most terrestrial ecosystems, with over 1600 genera and more than 34,000 species [14,15]. Throughout the centuries, members of the *Asteraceae* family have played a significant role in medicine, reflecting their remarkable diversity and broad therapeutic potential [16]. Many well-known species continue to attract scientific interest due to their broad-spectrum healing properties and their extensive applications in medicine, functional foods, and cosmetic products. The plant species of Asteraceae produce a broad spectrum of biologically active compounds, including phenolic acids, flavonoids, terpenoids, and volatile components or acetylenes [15]. They exhibit strong antibacterial, anti-inflammatory, antioxidant, anticancer, and antiparasitic activities [14], emerging as a promising source of bioactive compounds suitable for the development of novel therapeutics targeting neglected protozoan diseases [17]. In this context, *Argyranthemum* is a genus comprising approximately 22–24 species of flowering plants in the Asteraceae family, commonly known as marguerite or marguerite daisies. The genus is endemic to the Macaronesian region of the North Atlantic, which includes the archipelagos of the Canary Islands, the Azores, Madeira, Cape Verde and the Savage Islands. Several species, particularly *A. frutescens,* are widely cultivated as ornamental varieties. Furthermore, Argyranthemum species are known to predominantly biosynthesise polyacetylene and polyyne compounds. These compounds are widely distributed in nature, exhibiting biochemical and ecological functions, as well as a broad spectrum of biological and pharmacological activities and substantial economic potential. To the best of our knowledge, few studies have investigated the biological properties of *A. frutescens* [18,19,20], and none have examined its potential antiprotozoal effects. As part of our ongoing efforts to identify new antiparasitic agents from land plant diversity in Macaronesia, the roots of *A. frutescens* were selected for the present study. The inhibitory effect on *T. cruzi* and *Leishmania* spp. was then used in a bioactivity-guided approach to identify antiparasitic compounds in *A. frutescens*. Using this strategy, four bioactive acetylenes were isolated. The most active compound was selected for a preliminary mechanistic study.

## 2. Materials and Methods

### 2.1. Identification, Culture and Preparation of Plant Material

*Argyranthemum frutescens* (L.) was cultivated at La Tahonilla, Cabildo Insular de Tenerife, San Cristóbal de La Laguna, Tenerife, Canary Islands, Spain, in July 2023, following the protocol previously described by Dr. Cristina Montelongo, and a voucher specimen (TFG-54141) was deposited in the Herbarium TFG-SEGAI, Universidad de La Laguna (Tenerife, Spain). The roots were carefully spread out on a tray, turned periodically, and air-dried at room temperature (22 ± 4 °C) for two weeks. After drying, the roots were ground into a fine powder and stored for extraction.

### 2.2. Extract Preparation

The dried ground roots of *A. frutescens* (427.5 g) were extracted using a Soxhlet apparatus with 4 L of ethanol (EtOH). The extract was then filtered, and a rotary evaporator under reduced pressure was used to concentrate it, yielding 34.3 g of crude extract (8.0%).

### 2.3. Bioactivity-Guided Fractionation 

The crude extract was suspended in 450 mL of water and was subjected to liquid–liquid partition using solvents of increasing polarity: hexane (3 x 450 mL) and ethyl acetate (EtOAc) (3 x 450 mL). The organic extracts were concentrated under vacuum using a rotary evaporator, while the aqueous phase was freeze-dried, affording the hexane (Hex, 6.9 g), ethyl acetate (EtOAc, 5.3 g) and water (H_2_O, 18.5 g) fractions. The bioactive hexane fraction (6.9 g) was chromatographed on a silica gel column using a mixture of hexane/EtOAc (500 mL) of increasing polarity (10:0, 9:1, 8:2, 7:3, 6:4, 4:6, 0:10) and methanol as eluent to afford 8 subfractions, which were subsequently combined based on their thin-layer chromatography (TLC) profile into subfractions A1–A5. Biological evaluation of those subfractions revealed that subfraction A1 was the most active against *L. amazonensis*, *L. donovani*, and *T. cruzi*. Therefore, subfraction A1 (1.8 g) was chromatographed on Sephadex LH-20 (Hexane/CHCl_3_/MeOH, 2:1:1), affording subfractions A1A–A1C. Successive chromatographic separations were performed on the active subfraction, enabling the isolation and purification of the bioactive metabolites. Subfraction A1C (174.4 mg) was chromatographed on a silica gel column using a mixture of hexane/EtOAc of increasing polarity (10:0 to 7:3) as eluent to afford 32 subfractions, which were subsequently combined based on their thin-layer chromatography (TLC) profile into five subfractions (A1C1 to A1C5). An aliquot of subfraction A1C2 (11.4 mg) was further purified by preparative thin-layer chromatography (PTLC) using hexane/acetone (9:1) as eluent to afford compounds 1 (5.7 mg) and 2 (3.8 mg). An aliquot of subfraction A1C4 (16.0 mg) was further purified by PTLC using hexane/CH_2_Cl_2_ (10:0.5) as the mobile phase to afford compounds 3 (2.1 mg) and 4 (4.9 mg). The isolated compounds were characterised based on spectroscopic data and a comparison with reports in the literature and were identified as capillin (1) [21], 3′-*O*-demethyl frutescin (2) [22], capillinol (3) [21], and 3′-*O*-demethyl frutescinol acetate (4) [20].

### 2.4. Strains

Promastigote forms of *L. amazonensis* (MHOM/BR/77/LTB0016) and *L. donovani* (MHOM/IN/90/GE1F8R) were cultured in Schneider’s medium supplemented with 10% foetal bovine serum (FBS) at 26 °C. For the trypanocidal assay, the epimastigote form of *T. cruzi* (Y strain) was used, cultured in liver infusion tryptose (LIT) medium supplemented with 10% foetal bovine serum (FBS) at 26 °C. Murine macrophage cell line J774A.1 (ATCC #TIB-67) was maintained in DMEM at 37 °C in a 5% CO_2_ atmosphere for cytotoxicity assays.

### 2.5. In Vitro Antiparasitic Activity Against Leishmania spp. And T. cruzi

To test the activity against promastigote forms of *Leishmania* spp. or epimastigote forms of *T. cruzi*, serial dilutions of the compounds were prepared in 96-well microplates in a final volume of 100 µL with 100 µL of parasites at 10^6^ parasites/mL. After 48 h of incubation at 26 °C, 10% alamarBlue™ cell viability reagent (Thermo Fisher Scientific, Waltham, MA, USA) was added. Finally, the fluorescence of each well was measured 24 h later using an EnSpire Multimode Plate Reader^®^ (PerkinElmer, Thermo Fisher Scientific, Madrid, Spain). The half-maximal inhibitory concentration (IC_50_) was calculated using nonlinear regression analysis [23].

### 2.6. In Vitro Antiparasitic Activity Against the Amastigote Stage

To determine the IC_50_ against intracellular forms of the parasites, the amastigote forms of *L. amazonensis* and *T. cruzi* were used, and the alamarBlue™ colorimetric method was also developed. In a 96-well plate, murine macrophages were introduced at a concentration of 10^5^ cells/mL. After cell attachment, parasites were added at a concentration of 10^6^ parasites/mL to achieve a 1:10 cell-to-parasite ratio per well. The plates were incubated at 37 °C with 5% CO_2_ for 24 h to allow parasite internalization. The wells were then washed and treated with serial dilutions of the tested compounds. After 24 h of incubation with the compounds, the wells were washed, and the macrophages were lysed with 0.05% SDS (sodium dodecyl sulfate, 30 µL/well) for 30 s with agitation. Following this, 170 µL of medium was quickly added to stop the action of the SDS, preventing it from damaging the parasites, and the results were always compared with a negative control that had not been treated. Finally, after 72 h of incubation at 26 °C, 10% alamarBlue™ was added. The fluorescence was measured 24 h later using an EnSpire^®^ multi-mode plate reader, and the IC_50_ value of the compound was calculated [24].

### 2.7. Cytotoxicity Activity

The cytotoxic concentration 50 (CC_50_) was determined using the same alamarBlue™ colorimetric assay. For this assay, 50 μL of the murine macrophage suspension in RPMI medium (2 × 10^5^ cells/mL) was introduced into each well of a 96-well plate. After the cells had fully adhered, serial dilutions of the pure compounds, previously prepared in a deep-well plate, were added with 10% alamarBlue™. After 24 h of incubation at 37 °C and 5% CO_2_, fluorescence was evaluated using the EnSpire^®^ multi-mode plate reader at an excitation wavelength of 544 nm and an emission wavelength of 590 nm. The selectivity index (SI) for each compound was calculated as the ratio CC_50_/IC_50_ [25].

### 2.8. Mechanisms of Cell Death

To investigate the different mechanisms of action involved in the parasites, 200 µL of 10^6^ parasites/mL, comprising the epimastigote form for the *T. cruzi* assay and the promastigote form for the *L. amazonensis* assay, was incubated in 96-well plates with the previously determined IC_90_ of compound (**1**). After 24 hat 26 °C, the parasites were centrifuged at 3000 rpm for 10 min, and the pellet was resuspended in buffer for the addition of the different commercial kits.

#### 2.8.1. Chromatin Condensation Analysis

Chromatin condensation was detected using Hoechst 33342 at a concentration of 5 µg/mL. As stated by the manufacturer, this reagent enables the observation of fluorescence in condensed chromatin; therefore, cells exhibiting a high degree of chromatin condensation will emit more intense fluorescence. The parasites were incubated for 20 min at 26 °C in the dark. Fluorescence was examined using an EVOS M5000 Imaging System (Invitrogen, Thermo Fisher Scientific, Madrid, Spain) with a DAPI (excitation 350 nm/emission 461 nm) light cube [26].

#### 2.8.2. Plasma Membrane Permeability

To detect changes in membrane permeability in parasites, the SYTOX^®^ Green assay was used. This reagent is unable to pass through cell membranes when they are healthy; however, when the permeability of the membrane is altered, even if only by small pores, it is able to enter the cell, binding to nucleic acids and emitting fluorescence. After incubation with the IC_90_, SYTOX® Green was added at a final concentration of 1 μM. Finally, the presence of fluorescence was measured using the EVOS M5000 Imaging System with the GFP light cube (excitation 504 nm/emission 523 nm) [27].

#### 2.8.3. Mitochondrial Membrane Potential Analysis

The decrease in mitochondrial membrane potential was measured using the JC-1 Mitochondrial Membrane Potential Assay Kit (Cayman Chemical, Michigan, MI, USA), a dye that can selectively enter mitochondria and change color from green to red as the membrane potential increases. After incubation, the pellet was resuspended in 50 µL of buffer and transferred to a black 96-well plate, followed by the addition of 5 μL of JC-1 dye. After 30 min, fluorescence intensities for both green (monomeric form; 485/535 nm) and red (aggregate form; 535/590 nm) JC-1 were measured in the EnSpire Multimode Plate Reader^®^ [28].

#### 2.8.4. Measurement of ATP Levels 

Intracellular ATP levels were measured using the CellTiter-Glo^®^ Luminescent Cell Viability Assay (Promega Biotech Ibérica S.L., Madrid, Spain), as this reagent emits a luminescent signal that is directly proportional to the amount of ATP present in the sample. After incubation, the pellet was resuspended in 100 µL of buffer and transferred to a white 96-well plate, followed by the addition of 100 μL of the kit. After 2 min of agitation and 15 min of incubation, the luminescence was measured using the EnSpire Multimode Plate Reader^®^ [29].

#### 2.8.5. Detection of Reactive Oxygen Species

Cellular oxidative stress was assessed by detecting reactive oxygen species (ROS) using CellROX^®^ Deep Red reagent (Thermo Fisher Scientific, Waltham, MA, USA), as this reagent is not fluorescent in its reduced state but exhibits fluorescence following oxidation by reactive oxygen species. After incubation and centrifugation, the pellet was resuspended in 50 μL of assay buffer with CellROX^®^ reagent at a final concentration of 5 μM. Finally, fluorescence images were captured using an EVOS M5000 Imaging System in the Cy5 light cube [30].

### 2.9. Statistical Analysis

The assays were performed in triplicate on three different days. The IC_50_ and CC_50_ were calculated using nonlinear regression with 95% confidence intervals in GraphPad PRISM^®^ 10.1.1 software from GraphPad Software in San Diego, CA, USA. The results are expressed as the mean of the three replicates, with the standard deviation (SD). Plots were also created using GraphPad PRISM^®^ 10.1.1. One-way ANOVA was used to assess statistical significance. Differences with *p* < 0.05 were considered statistically significant. Quantitative image analysis was performed without blinding; however, potential sources of bias were mitigated by the application of predefined evaluation criteria and uniform imaging conditions across all treatments. A total of three images per experiment, derived from independent experimental replicates conducted on three separate days, were subjected to analysis. At least 200 cells were counted in each image, making a total of at least 600 cells per compound.

## 3. Results and Discussion

### 3.1. Bioassay-Guided Fractionation

Bioactivity-guided fractionation is a powerful phytochemical analysis tool in natural drug discovery, offering a strategic pathway towards developing safe and effective disease management therapies. In this framework, bioassay-guided fractionation of crude herbal extracts represents a highly promising and rational approach within natural product-based drug development programs, enabling the systematic isolation and characterization of bioactive compounds responsible for therapeutic effects [31].

In this study, as part of our research program aimed at discovering plant-derived antikinetoplastid agents, we carried out a bioassay-guided fractionation of the ethanolic extract from cultivated *A. frutescens* roots. The *in vitro* 50% inhibitory concentration (IC_50_) of the extract, fractions, and subfractions was determined against different kinetoplastid species, including the extracellular stages of *L. amazonensis*, *L. donovani*, and *T. cruzi*. Commercial antikinetoplastid agents, benznidazole and miltefosine, were employed as reference drugs for their trypanocidal and leishmanicidal activities, respectively, to enable comparative assessment in the present analysis.

**Figure 1:**
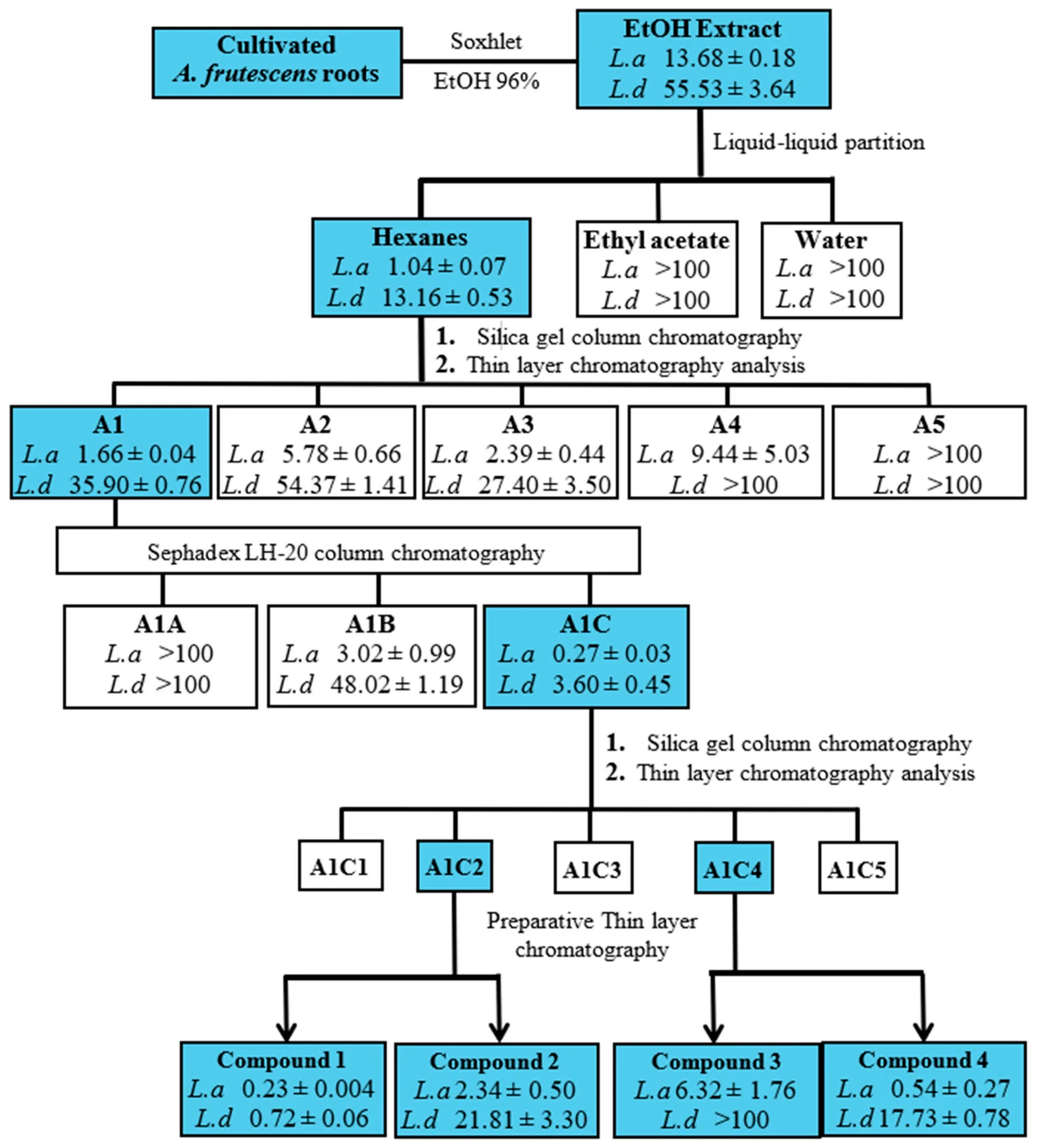
The flowchart shows the bio-guided fractionation of the root extract of *A. frutescens* against promastigotes of *L. amazonensis* (*L.a*) and *L. donovani* (*L.d*). Leishmanicidal activity is expressed as the half-maximal inhibitory concentration (in µg/mL). The values are expressed as mean ± SD. Miltefosine (IC_50_ of 2.64 µg/mL against *L. amazonensis,* and IC_50_ of 1.35 µg/mL against *L. donovani*) was used as a positive control.

**Figure 2:**
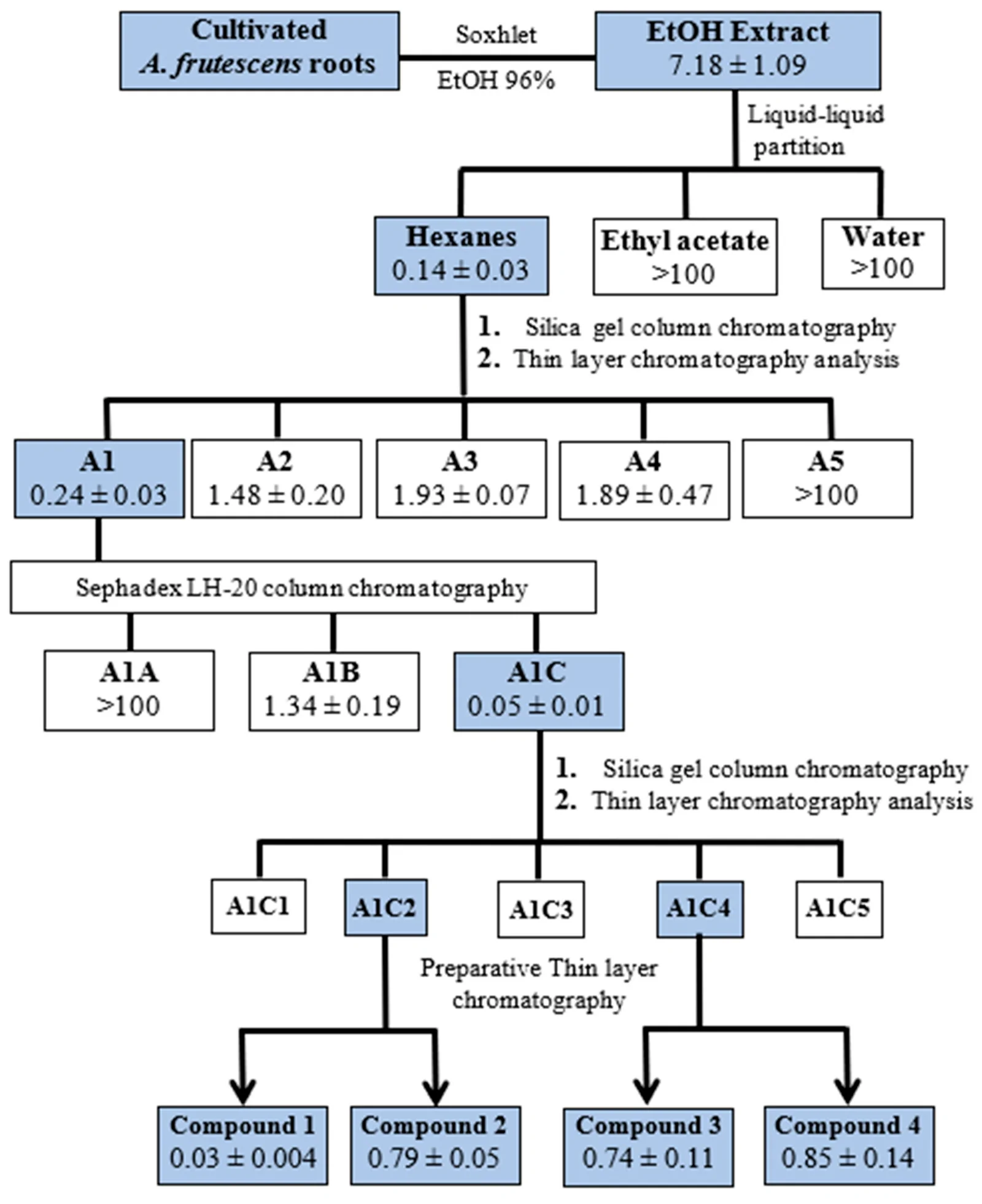
The flowchart shows the bio-guided fractionation of the root extract of *A. frutescens* against *T. cruzi* epimastigotes. Trypanocidal activity is expressed as mean ± SD in µg/mL. Benznidazole (IC_50_, 1.80 µg/mL) was used as a positive control.

The ethanolic extract of the roots of *A. frutescens* was prepared using a Soxhlet apparatus. The obtained crude extract exhibited activity against *T. cruzi*, *L. amazonensis* and *L. donovani,* with IC_50_ values of 7.18 μg/mL, 13.68 μg/mL and 53.53 μg/mL, respectively (Figure 1 and Figure 2).

These positive results suggested that a bioassay-guided fractionation should be carried out. Therefore, the EtOH extract was successively fractionated by liquid–liquid partitioning using other solvents with increasing polarity to yield the corresponding hexane (Hex), ethyl acetate (EtOAc) and water (H_2_O) fractions. Remarkably, only the Hex fraction exhibited biological activity against *T. cruzi* (IC_50_ 0.14 μg/mL), *L. amazonensis* (IC_50_ 1.04 μg/mL) and *L. donovani* (IC_50_ 13.16 μg/mL) (Figure 1 and Figure 2).

**Figure 3:**
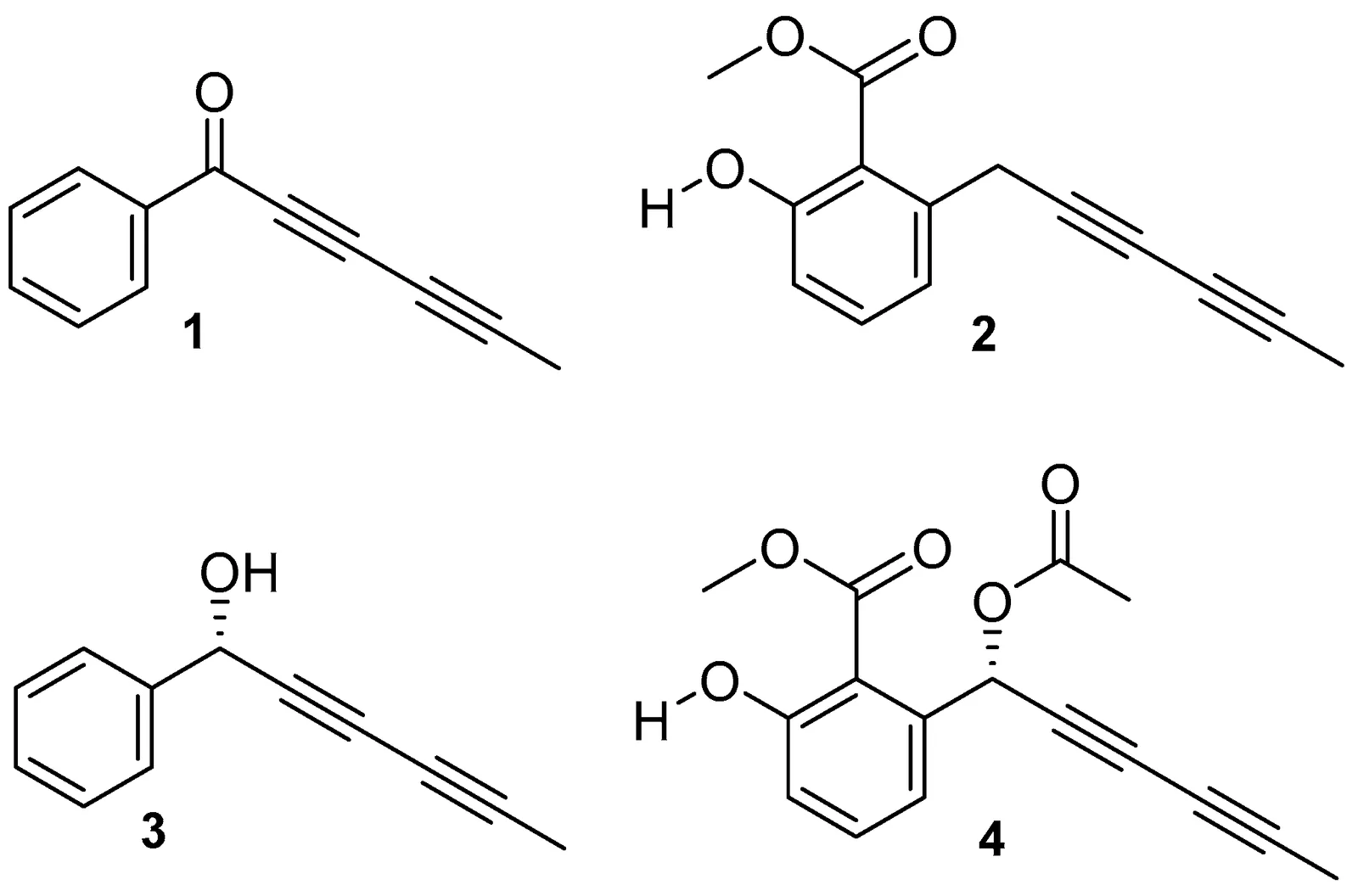
Chemical structures of polyacetylenes (**1**–**4**) isolated from bio-guided fractionation of cultivated *A. frutescens* root extract. The compounds were identified as capillin (**1**), 3′-O-desmethylfrutescin (**2**), capillinol (**3**) and 3′-O-desmethylfrutescinol acetate (**4**).

Moreover, the hexane (Hex) fraction exhibited stronger activity against *L. amazonensis* than the commercial leishmanicidal agent miltefosine (IC_50_ = 2.64 µg/mL) and surpassed the activity of the trypanocidal reference drug benznidazole (IC_50_ = 1.80 µg/mL). It is worth noting that this fraction exhibited greater antikinetoplastid activity than the crude extract against all parasites. Therefore, the Hex fraction emerged as the most promising candidate for further bioassay-guided fractionation. It was subsequently chromatographed on silica gel, yielding five subfractions (A1–A5). Evaluation of antiparasitic activity showed that subfractions A1–A4 exhibited potent effects (Figure 1 and Figure 2), with IC_50_ values ranging from 0.24 to 1.93 μg/mL against *T. cruzi* and from 1.66 to 9.44 μg/mL against *L. amazonensis*. Regarding *L. donovani*, subfractions A1–A3 showed moderate activity with IC_50_ values ranging from 27.40 to 54.37 μg/mL. According to these results, fraction A1 was the most active one, showing potent activity against all three parasite lines, although its overall biological profile was slightly less potent than that of the Hex fraction. Thus, based on bioactivity, subfraction A1 was selected for successive purification steps by column chromatography to identify the bioactive components. This subfraction was further fractionated into three subfractions (A1A–A1C) by size exclusion column chromatography on Sephadex LH-20. Subfractions A1B and A1C showed strong antiparasitic activity against *T. cruzi* and *L. amazonensis* and showed moderate activity against *L. donovani* (Figure 1 and Figure 2). Fraction A1C was the most active, showing potent activity against all three parasite lines, with IC_50_ values of 0.27 μg/mL against *L. amazonensis,* 3.60 μg/mL against *L. donovani* and 0.05 μg/mL against *T. cruzi*. Based on its antiparasitic activity profile, subfraction A1C (174.4 mg) was subjected to silica gel column chromatography, affording five fractions (A1C1–A1C5). Then, subfractions A1C2 and A1C4 were identified as the most promising candidates for compound isolation by TLC analysis and were subsequently purified by preparative thin-layer chromatography (PTLC), yielding compounds 1 and 2 from A1C2, and compounds 3 and 4 from A1C4 (Figure 1, Figure 2 and Figure 3). Bioassay-guided fractionation of the active fractions led to the isolation of four compounds, identified as capillin (1) [21], 3′-*O*-desmethylfrutescin (2) [22], capillinol (3) [21] and 3′-*O*-desmethylfrutescinol acetate (4) [20].

### 3.2. Antikinetoplastid Activity of the Isolated Compounds

Once the bio-guided fractionation process was completed and the major constituents of the active subfractions were successfully isolated, these compounds (**1**–**4**) were assayed for their *in vitro* kinetoplastid activity against different forms of *T. cruzi*, *L. donovani* and *L. amazonensis*. Additionally, the 50% cytotoxic concentrations (CC_50_) of the compounds were evaluated using a eukaryotic cell line (murine macrophage cell line J774A.1). Compounds were further classified based on safety criteria [32]. Compounds that exhibited a selectivity index (SI) ≥ 10 are typically regarded as possessing satisfactory selective toxicity, indicating a preferential inhibition of the parasite relative to host mammalian cells. Benznidazole and miltefosine were employed as reference drugs for their trypanocidal and leishmanicidal activities, respectively.

The trypanocidal assays demonstrated that all compounds (**1**–**4**) exhibited pronounced activity in the low micromolar range, with IC_50_ values between 0.11 and 4.10 µM, showing higher potency than the reference drug (Table 1). Among them, compound 1 emerged as the most active compound, displaying 63.1-fold higher potency than that of the positive control. Moreover, three of them (**1**–**3**) displayed good to outstanding selectivity. Remarkably, compound 1 exhibited an exceptional selectivity index (SI) of 1815.5, vastly surpassing the SI of the reference drug (SI = 57.6), thereby highlighting its pronounced preferential toxicity toward the parasite over host cells.

The leishmanicidal activity revealed pronounced differences among the tested species (Table 2). In general, the compounds displayed a broad yet variable activity profile, with *L. amazonensis* being notably more susceptible to the evaluated polyacetylenes than *L. donovani*. Compound **1** was the most active agent (IC_50_ = 0.70 µM), showing a nine-fold increase in potency over miltefosine against *L. amazonensis*. Compounds 2 (IC_50_ = 8.1 µM) and 4 (IC_50_ = 3.8 µM) exhibited comparable activity to the reference drug, while compound 3 was only weakly active. The noteworthy potency of compound 1, coupled with its high selectivity (SI = 285), underscores its excellent pharmacological prospects. The activity of the polyacetylenes against *L. donovani* was clearly reduced, with all compounds showing lower potency and selectivity than the control drug. This pattern suggests species-dependent differences in sensitivity to these compounds.

Based on a combined analysis of the antikinetoplastid activity and cytotoxicity, compound **1** stood out as the most promising candidate for further development, owing to its favourable profile against *T. cruzi* and *L. amazonensis*. Therefore, we proceeded to assess its antiparasitic potential against the intracellular amastigote stages of these protozoa. Compound **1** showed higher potency than the positive control against *L. amazonensis* amastigotes, with an IC_50_ value of 1.70 ± 0.08 µM compared with 3.12 ± 0.30 µM for miltefosine. On the other hand, its activity against *T. cruzi* amastigotes was lower than that of benznidazole, with IC_50_ values of 5.30 ± 0.20 and 2.67 ± 0.39 µM, respectively. Compound 1 showed a CC_50_ value of 199.7 ± 3.37 µM against macrophages, resulting in selectivity indices of 117.5 and 37.7 against *L. amazonensis* and *T. cruzi*, respectively. These values were higher than those obtained for miltefosine (SI = 23.1) against *L. amazonensis*, although lower than those obtained for benznidazole (SI = 149.8) against *T. cruzi* (Table 3). Despite the well-documented adverse effects associated with benznidazole, including dermatological, gastrointestinal, and neurological manifestations [33], capillin emerges as a promising candidate with antikinetoplastid activity.

Indeed, this polyacetylene, which is primarily found in *Artemisia capillaris*, has been reported to exhibit a variety of biological activities, including antitumour, antimicrobial and antifungal effects [34].

### 3.3. Analysis of Cell Death MechanismsIinduced by Capillin in L. amazonensis and T. cruzi

**Figure 4:**
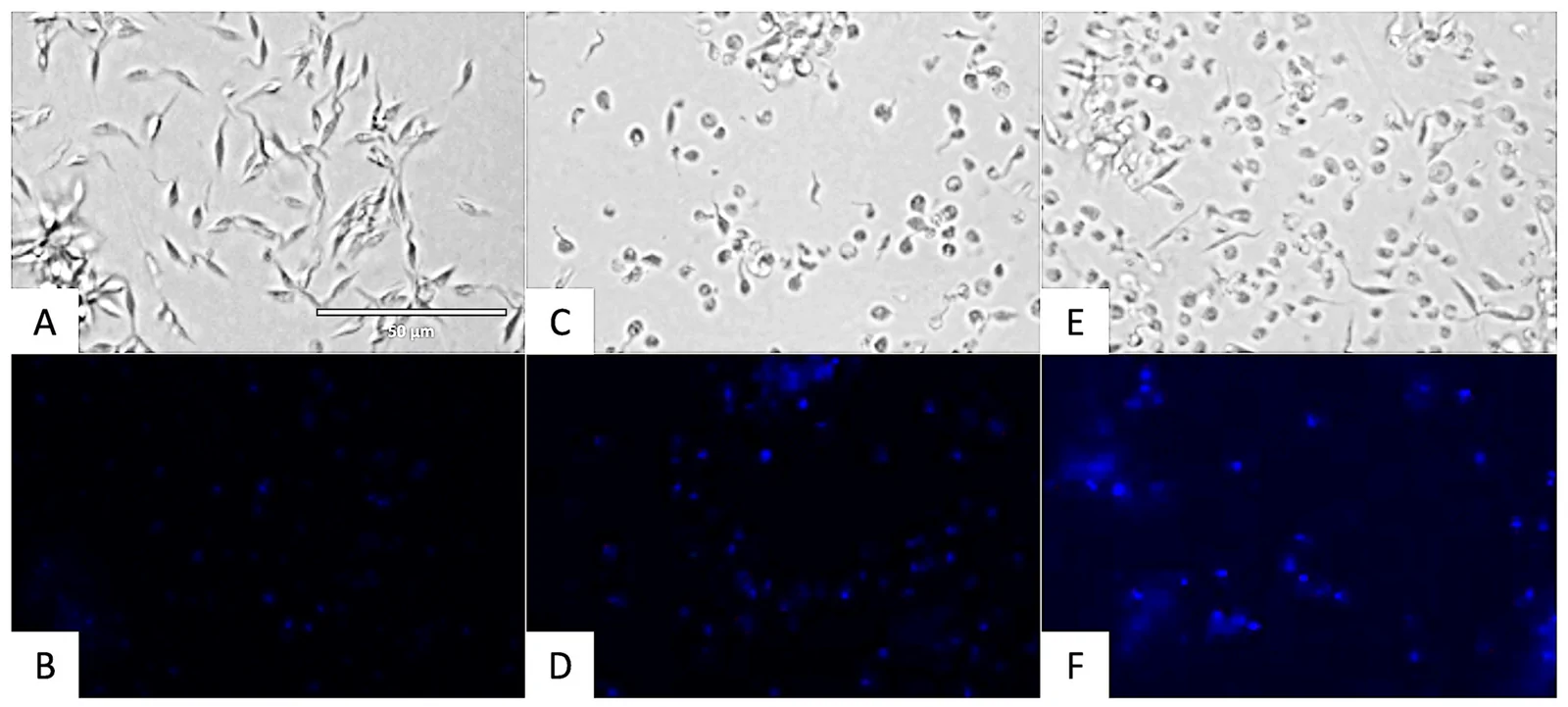
Representative images of *L. amazonensis* stained with Hoechst and acquired using the EVOS M5000 Imaging System (40×) after 24 h of incubation at 26 °C. (**A**) Untreated parasites observed in the visible channel. (**B**) Untreated parasites in the DAPI channel. (**C**) Parasites treated with the IC_90_ of miltefosine in the visible channel. (**D**) Parasites treated with the IC_90_ of miltefosine in the DAPI channel. (**E**) Parasites treated with the IC_90_ of capillin in the visible channel. (**F**) Parasites treated with the IC_90_ of capillin in the DAPI channel. Scale bar: 50 µm.

**Figure 5:**
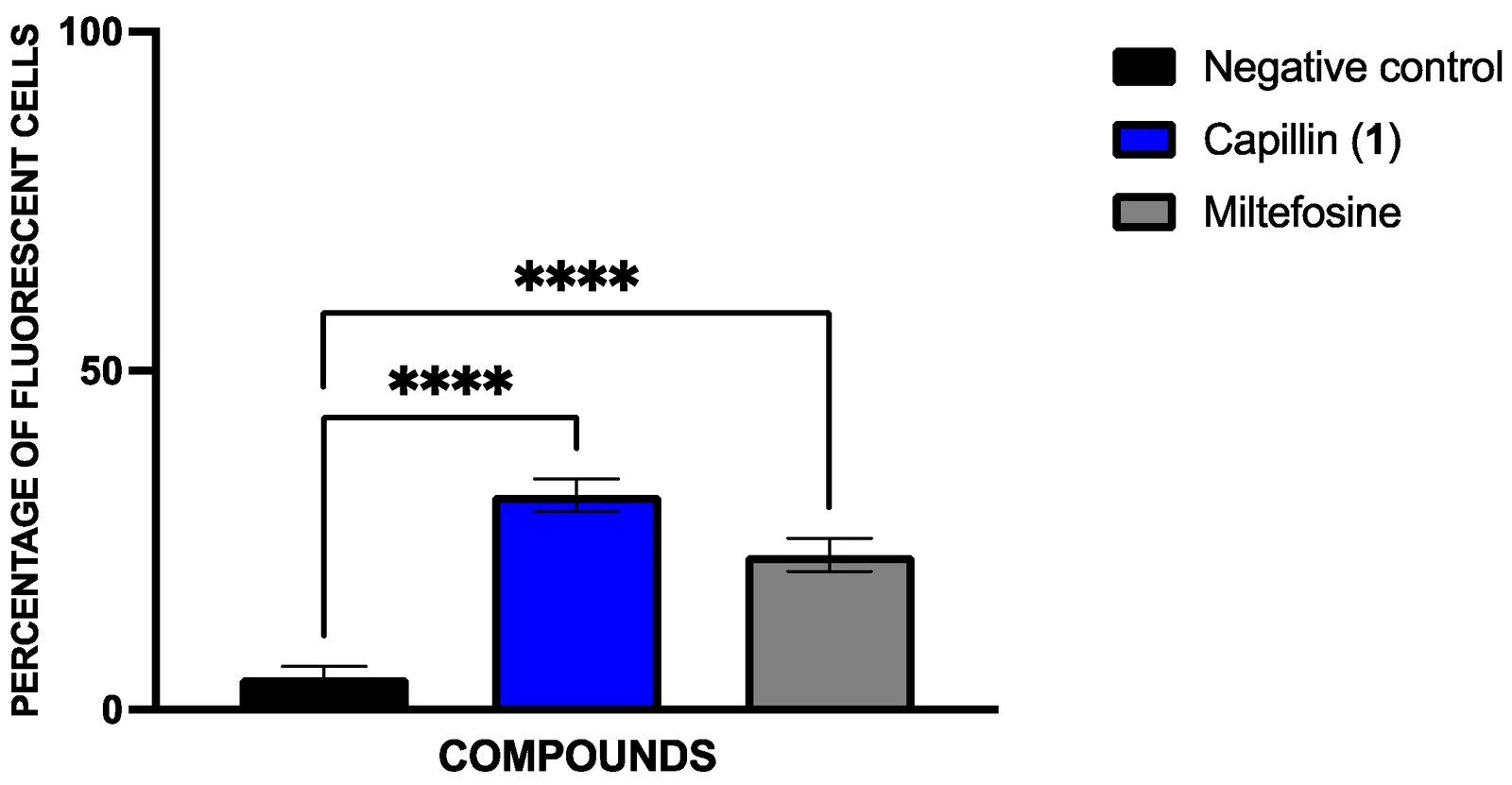
Quantification of the Hoechst assay in *L. amazonensis*. Parasites were manually counted using the EVOS M5000 Imaging System software. Miltefosine was included as the reference treatment. For each treatment, parasites were quantified from three independent replicate images, with at least 200 cells counted in each image. Statistical analysis was performed using a Tukey multiple-comparisons test in GraphPad Prism^®^ (version 10.1.1) to assess differences between treatment means relative to the negative control. Statistical significance was set at *p* < 0.0001 (****).

**Figure 6:**
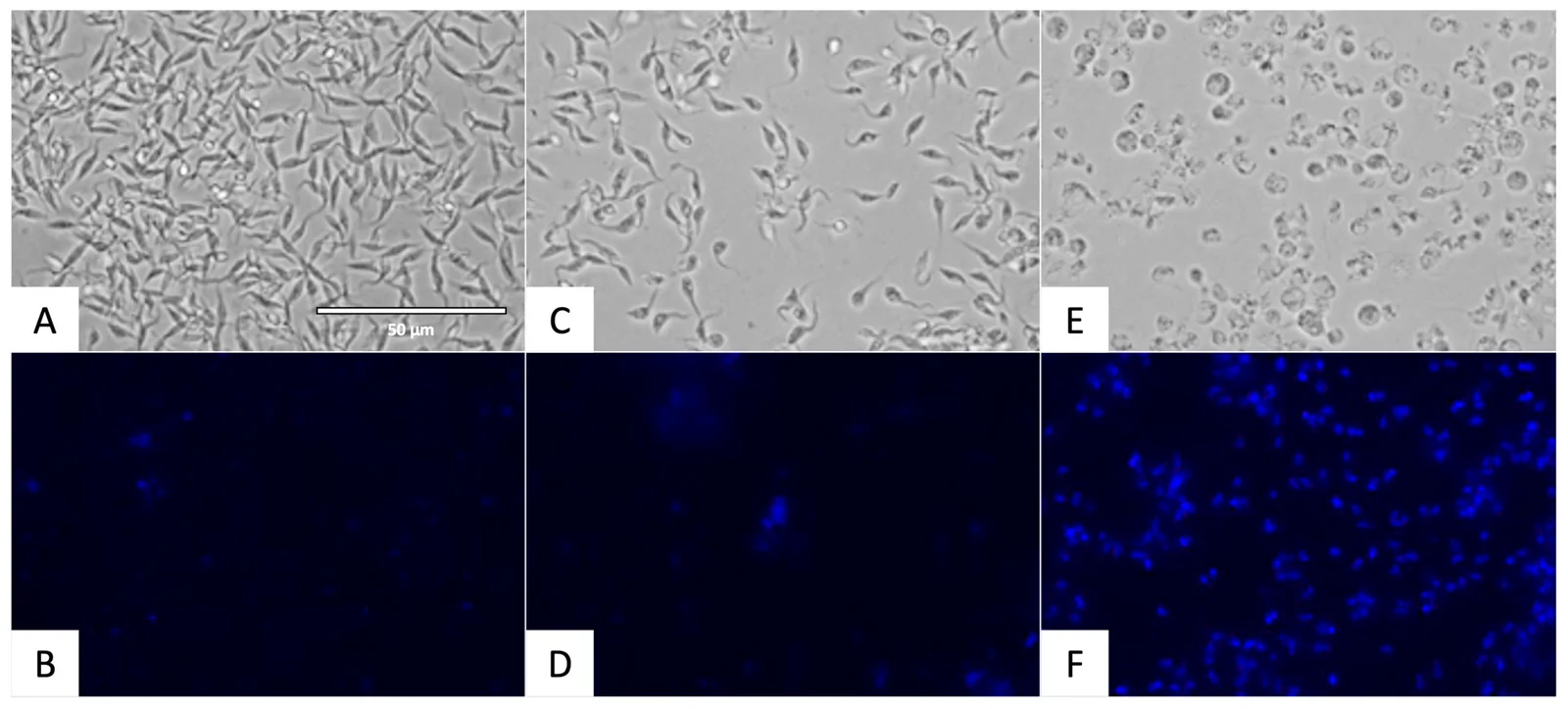
Representative images of *T. cruzi* stained with Hoechst and acquired using the EVOS M5000 Imaging System (40×) after 24 h of incubation at 26 °C. (**A**) Untreated parasites observed in the visible channel. (**B**) Untreated parasites in the DAPI channel. (**C**) Parasites treated with the IC_90_ of benznidazole in the visible channel. (**D**) Parasites treated with the IC_90_ of benznidazole in the DAPI channel. (**E**) Parasites treated with the IC_90_ of capillin in the visible channel. (**F**) Parasites treated with the IC_90_ of capillin in the DAPI channel. Scale bar: 50 µm.

**Figure 7:**
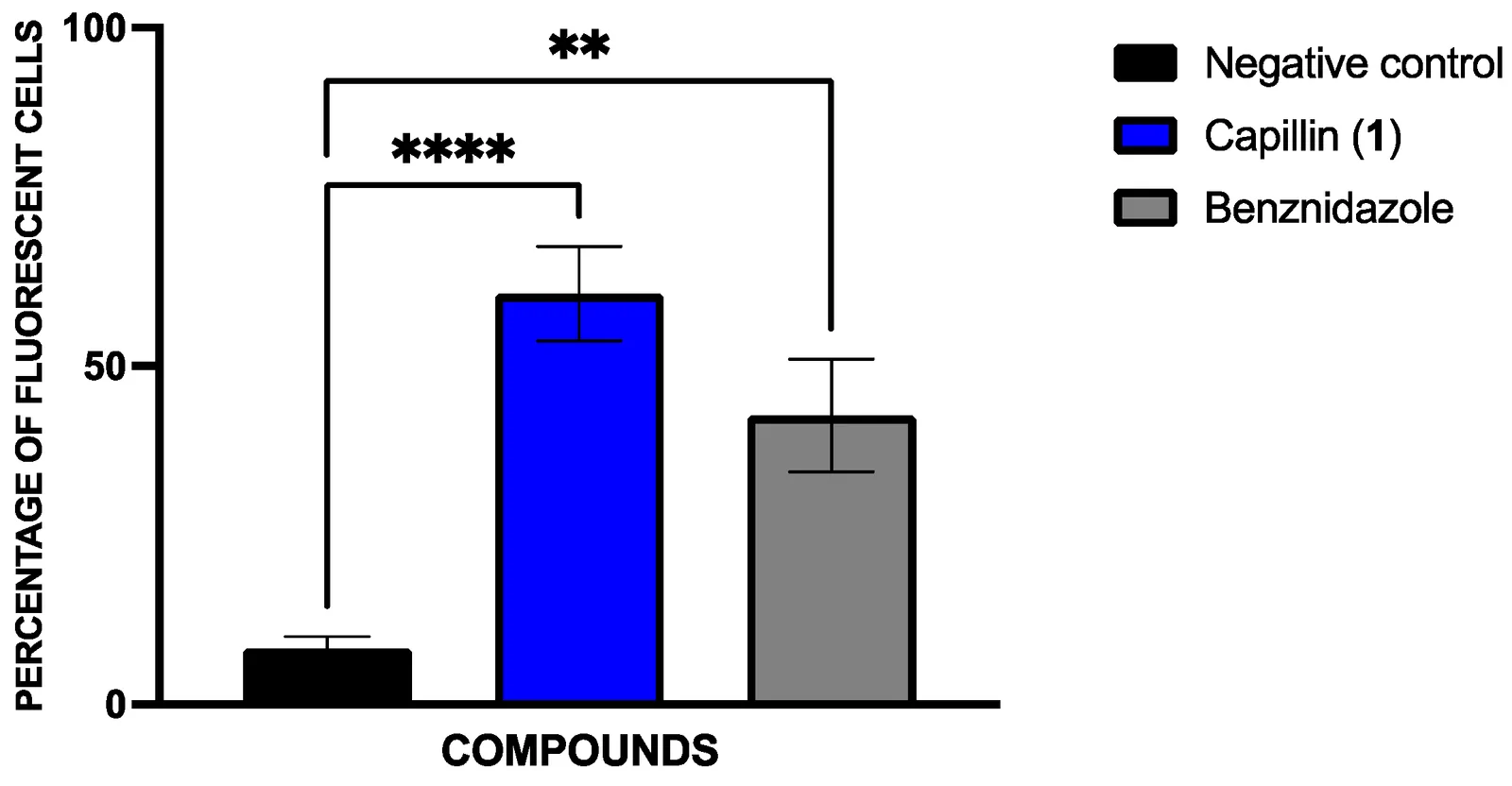
Quantification of the Hoechst assay in *T. cruzi*. Parasites were manually counted using the EVOS M5000 Imaging System software. Benznidazole was used as the reference treatment. For each treatment, parasites were quantified from three independent replicate images, with at least 200 cells counted in each image. Statistical analysis was performed using a Tukey multiple-comparisons test in GraphPad Prism^®^ (version 10.1.1) to assess differences between treatment means relative to the negative control. Statistical significance was set at *p* < 0.01 (**) and *p* < 0.0001 (****).

The *in vitro* antikinetoplastid activity and selectivity index observed for compound 1 provide sufficiently promising results to warrant further investigation of its potential mechanisms of action in the epimastigote or promastigote stages of *T. cruzi* and *L. amazonensis,* respectively. Accordingly, in the present study, different experiments were carried out to elucidate and characterize the effects of capillin on key cellular features, including DNA fragmentation, cell shrinkage, plasma membrane alterations, and mitochondrial dysfunction [35,36]. Furthermore, miltefosine and benznidazole were evaluated as positive controls for comparative analysis in *L. amazonensis* and *T. cruzi*, respectively, while untreated parasites were used as the negative control. Chromatin condensation stands out as one of the most characteristic markers of apoptotic cell death [37]. In protozoa, it has been recognized as a reliable indicator of an apoptosis-like process, as documented in species of the genera *Leishmania* and *Trypanosoma*, among others [38]. Hoechst 33342 reagent was used to detect chromatin condensation. In healthy cells, fluorescence appears weak and diffuse, whereas cells with condensed chromatin exhibit intense blue fluorescence. Hoechst dyes bind to the minor groove of double-stranded DNA [39]. During chromatin condensation, the increased DNA packing density enhances local dye concentration, resulting in higher fluorescence signal intensity [39,40]. Hoechst 33342 reagent was used to detect chromatin condensation in *L. amazonensis* promastigotes (Figure 4 and Figure 5) and *T. cruzi* epimastigotes (Figure 6 and Figure 7). The results showed that the cells exposed to compound **1** (capillin) in both parasites displayed markedly stronger blue fluorescence compared with the positive and negative controls (Figure 5 and Figure 7). These findings indicate that the compound remarkably enhances blue fluorescence in both parasites, suggesting a strong cellular response to the treatment. These data are consistent with previous results, in which the compound showed chromatin condensation in the HT29 colon carcinoma cell line [41].

**Figure 8:**
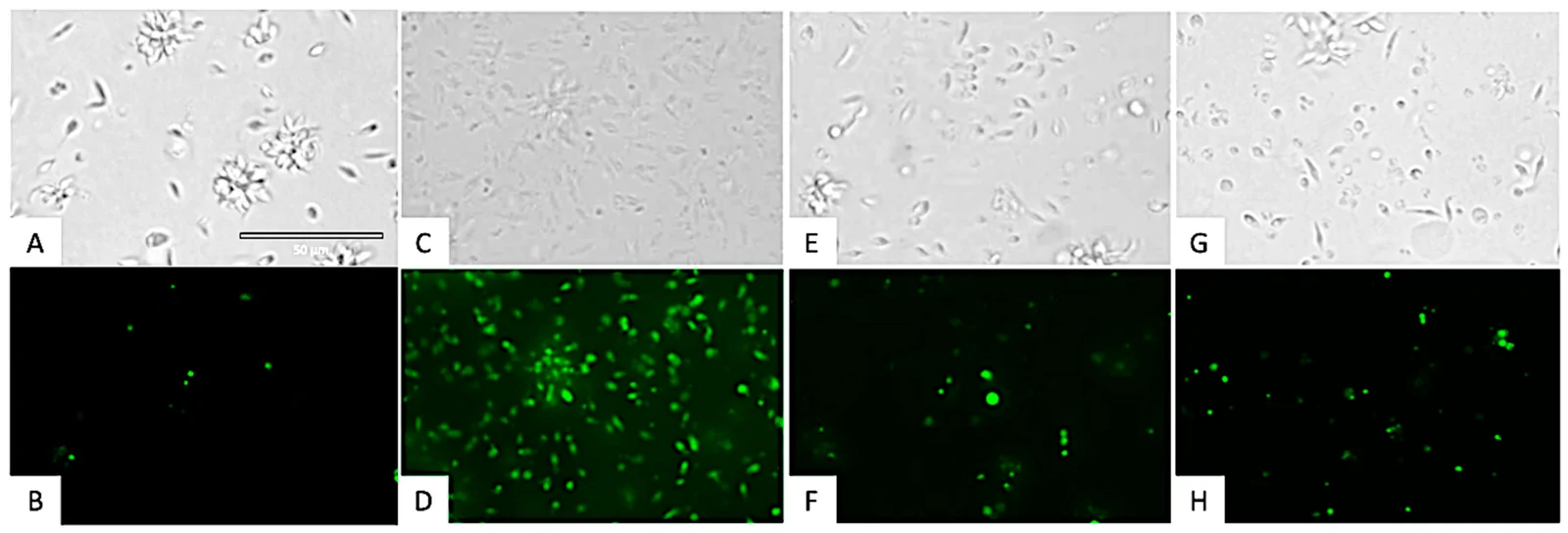
Representative images of *L. amazonensis* stained with SYTOX™ Green and acquired using the EVOS M5000 Imaging System (20×) after 24 h of incubation at 26 °C. (**A**) Untreated parasites observed in the visible channel. (**B**) Untreated parasites in the GFP channel. (**C**) Parasites treated with 0.1% Triton in the visible channel. (**D**) Parasites treated with 0.1% Triton in the GFP channel. (**E**) Parasites treated with the IC_90_ of miltefosine in the visible channel. (**F**) Parasites treated with the IC_90_ of miltefosine in the GFP channel. (**G**) Parasites treated with the IC_90_ of capillin in the visible channel. (**H**) Parasites treated with the IC_90_ of capillin in the GFP channel. Scale bar: 200 µm.

**Figure 9:**
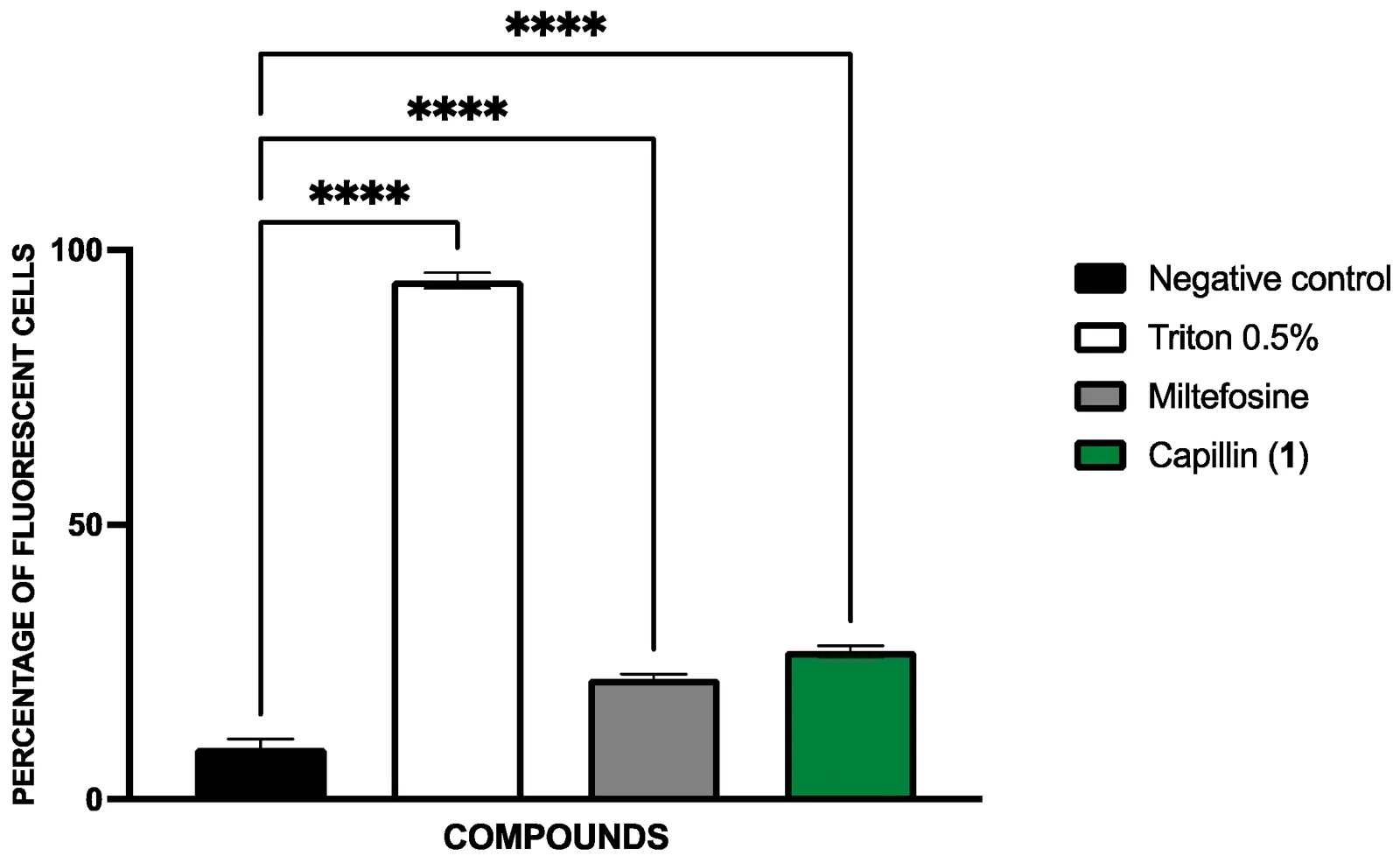
Quantification of the SYTOX™ Green assay in *L. amazonensis*. Parasites were manually counted using the EVOS M5000 Imaging System software. Miltefosine was included as the reference treatment and 0.5% Triton as the positive control. For each treatment, parasites were quantified from three independent replicate images, with at least 200 cells counted in each image. Statistical analysis was performed using a Tukey multiple-comparisons test in GraphPad Prism^®^ (version 10.1.1) to assess differences between treatment means relative to the negative control. Statistical significance was set at *p* < 0.0001 (****).

**Figure 10:**
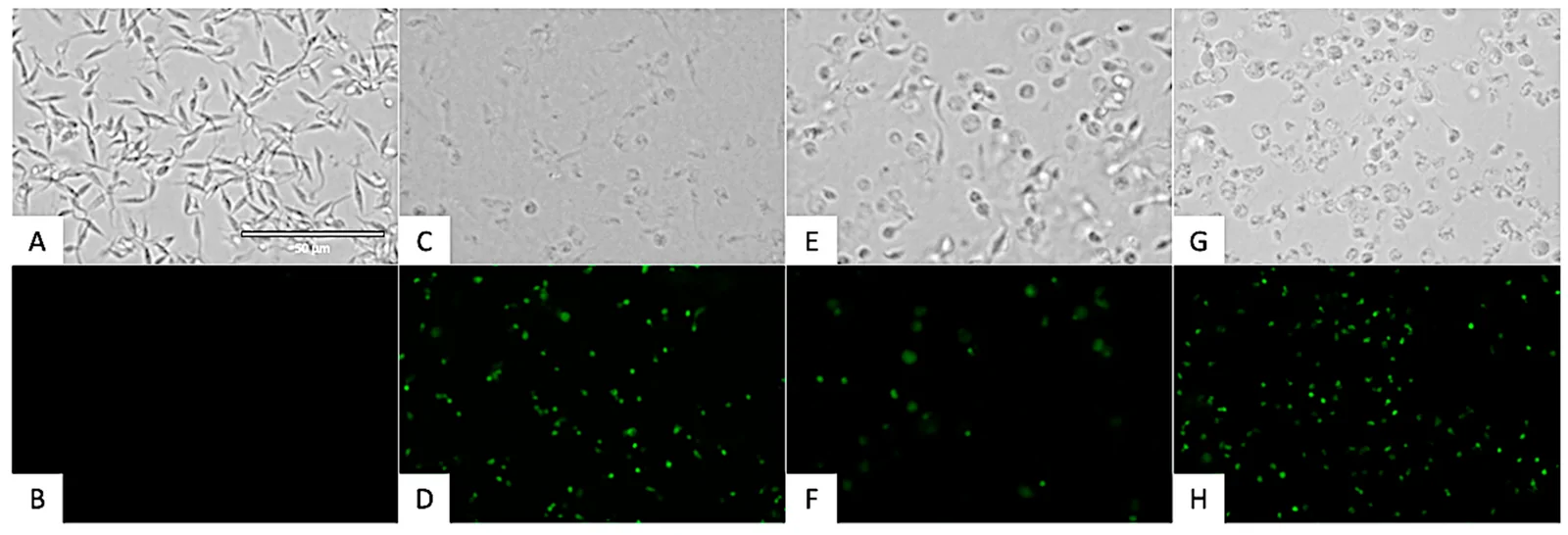
Representative images of *T. cruzi* stained with SYTOX™ Green and acquired using the EVOS M5000 Imaging System (20×) after 24 h of incubation at 26 °C. (**A**) Untreated parasites observed in the visible channel. (**B**) Untreated parasites in the GFP channel. (**C**) Parasites treated with 0.5% Triton in the visible channel. (**D**) Parasites treated with 0.1% Triton in the GFP channel. (**E**) Parasites treated with the IC_90_ of benznidazole in the visible channel. (**F**) Parasites treated with the IC_90_ of benznidazole in the GFP channel. (**G**) Parasites treated with the IC_90_ of capillin in the visible channel. (**H**) Parasites treated with the IC_90_ of capillin in the GFP channel. Scale bar: 200 µm.

**Figure 11:**
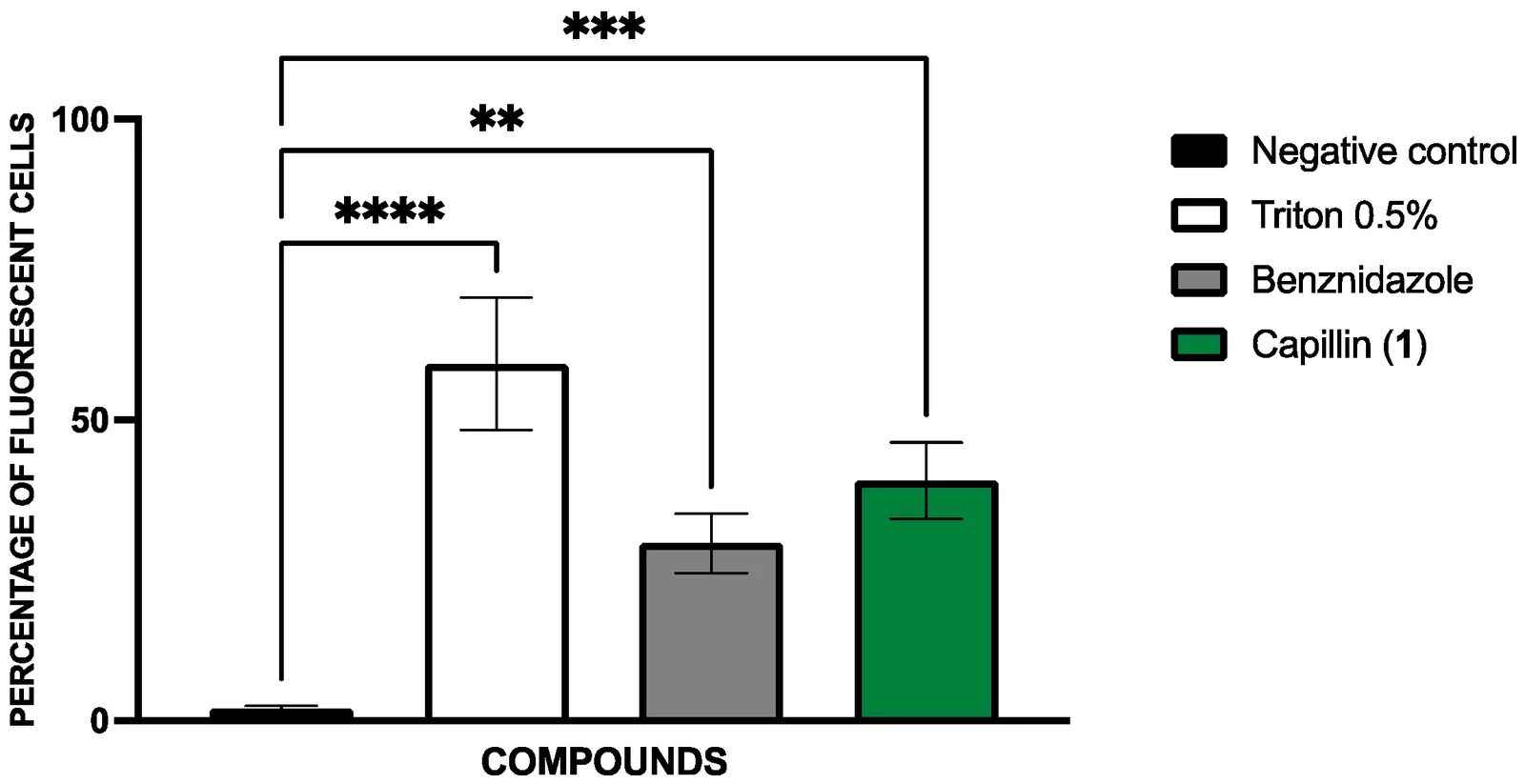
Quantification of the SYTOX™ Green assay in *T. cruzi*. Parasites were manually counted using the EVOS M5000 Imaging System software. Benznidazole was included as the reference treatment and 0.5% Triton as the positive control. For each treatment, parasites were quantified from three independent replicate images, with at least 200 cells counted in each image. Statistical analysis was performed using a Tukey multiple-comparisons test in GraphPad Prism^®^ (version 10.1.1) to assess differences between treatment means relative to the negative control. Statistical significance was set at *p* < 0.01 (**), *p* < 0.001 (***), and *p* < 0.0001 (****).

To evaluate the effect of capillin on cell membrane permeability, an experiment was carried out using SYTOX^®^ Green, a fluorochrome specific for nucleic acids (DNA/RNA), which is used as an indicator of cell viability and to assess the integrity of the plasma membrane in eukaryotic and prokaryotic cells. The dye penetrated only cells with damaged membranes, emitting a bright green fluorescence upon binding to nucleic acids, indicating loss of membrane integrity. The results on *L. amazonensis* (Figure 8 and Figure 9) and *T. cruzi* (Figure 10 and Figure 11) showed that the parasites exposed to capillin and the reference drugs displayed a marked increase in green fluorescence intensity relative to the negative control. The increase in fluorescence reflects a significant rise in plasma membrane permeability, indicating that these treatments adversely affect membrane integrity and consequently compromise parasite viability.

**Figure 12:**
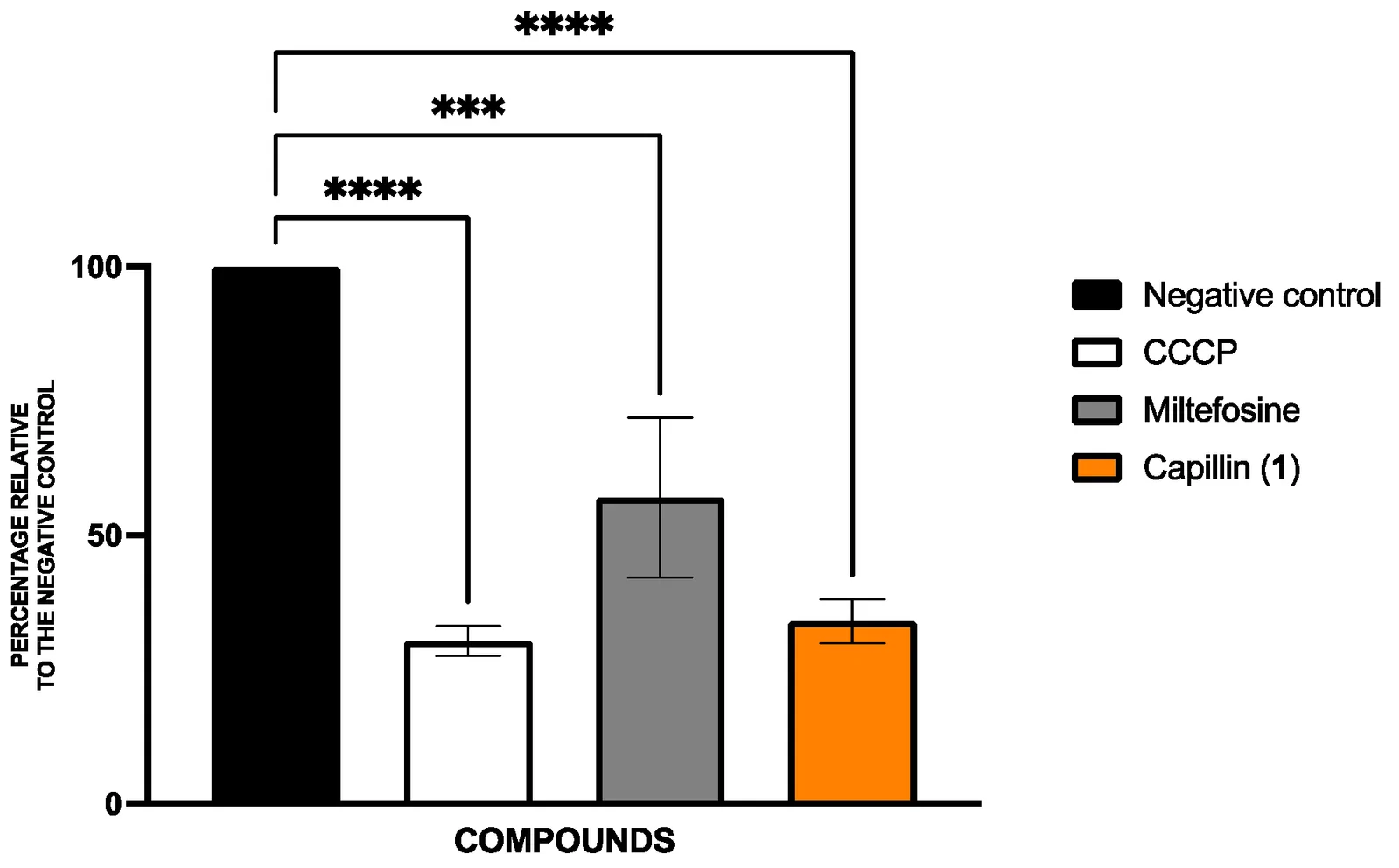
Quantification of the mitochondrial potential analysis with the JC-1 kit in *L. amazonensis*. The fluorescence was measured using the EnSpire Multimode Plate Reader^®^. Miltefosine was included as the reference treatment and carbonylcyanide-m-chlorophenylhydrazone (CCCP) as the positive control. For each treatment, parasites were quantified from three independent replicates, and a Tukey test was performed using GraphPad PRISM^®^ 10.1.1 software to assess statistical differences between means relative to the negative control. Statistical significance was set at *p* < 0.001 (***) and *p* < 0.0001 (****).

**Figure 13:**
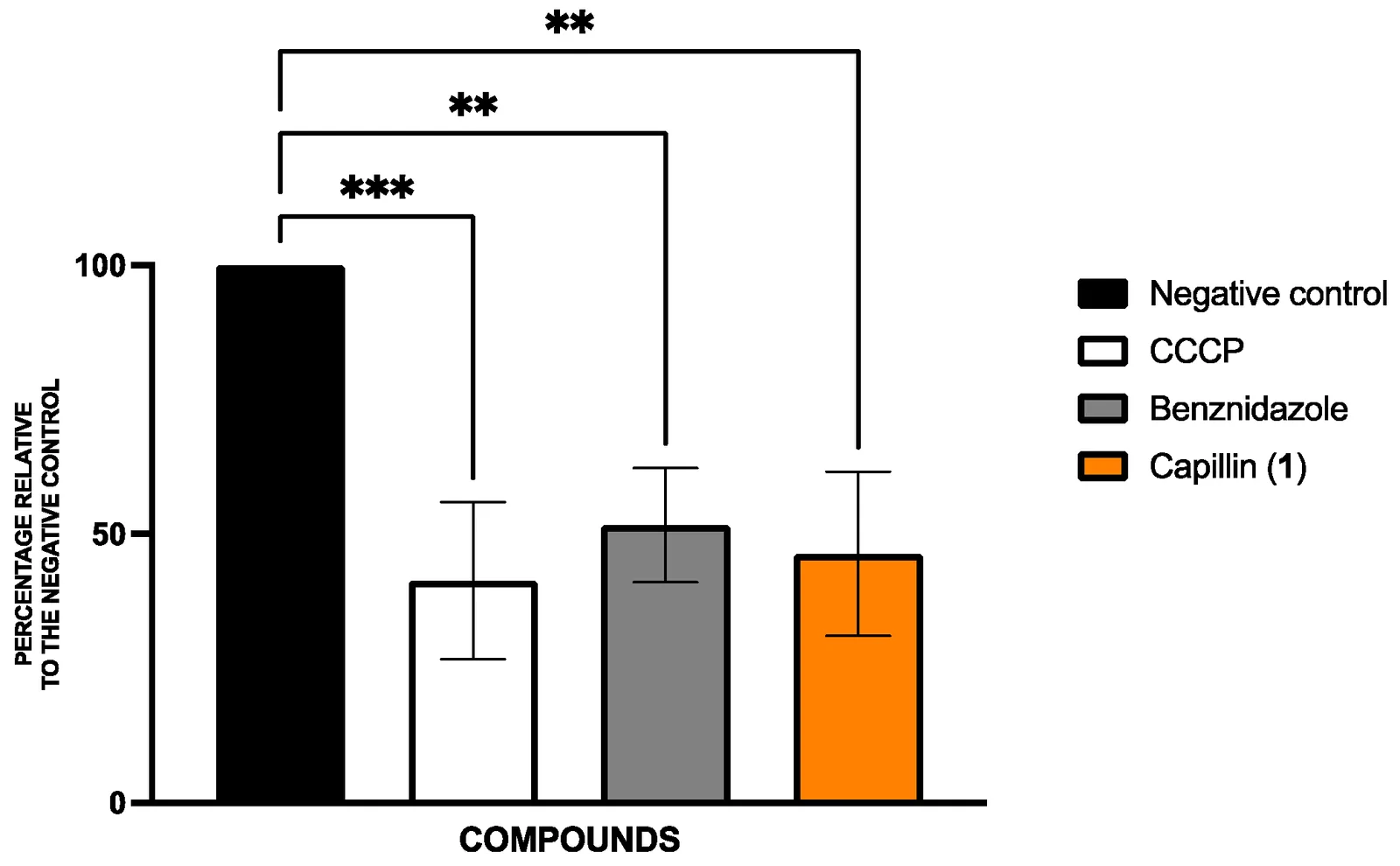
Quantification of the mitochondrial potential analysis with the JC-1 kit in *T. cruzi*. The fluorescence was measured using an EnSpire Multimode Plate Reader^®^. Benznidazole was included as the reference treatment and carbonylcyanide-m-chlorophenylhydrazone (CCCP) as the positive control. For each treatment, parasites were quantified from three independent replicates, and a Tukey test was performed using GraphPad PRISM^®^ 10.1.1 software to assess statistical differences between means relative to the negative control. Statistical significance was set at *p* < 0.01 (**) and *p* < 0.001 (***).

Being a small molecule (MW < 500 g/mol) and a lipophilic aromatic compound with an unsaturated side chain, capillin can interact with lipid bilayers, disrupting the membrane and increasing membrane fluidity/permeability [42]. Furthermore, mitochondrial damage is a key event in apoptosis, particularly within the intrinsic pathway of programmed cell death. This dysfunction is primarily associated with the collapse of the mitochondrial membrane potential. Moreover, impaired mitochondrial function promotes the excessive production of reactive oxygen species, which further contributes to the progression of programmed cell death [43,44]. In the current study, the effect of capillin on mitochondrial function was assessed by evaluating the mitochondrial membrane potential using the JC-1 dye, as well as intracellular ATP levels and reactive oxygen species (ROS) production. The effect of the isolated compound on mitochondrial membrane potential (ΔΨm) was evaluated using the JC-1 staining kit, revealing that the compound decreased ΔΨm by 50% in both parasites (Figure 12 and Figure 13). The mitochondrial membrane potential (ΔΨm) was quantified as percentages relative to the reference drugs (miltefosine for *L. amazonensis* and benznidazole for *T. cruzi*), the positive control (carbonylcyanide-*m*-chlorophenylhydrazone, CCCP), and the negative control (untreated parasites). These alterations in ΔΨm suggest mitochondrial dysfunction consistent with the induction of apoptosis in the parasite cells, a hallmark of treatment-induced cell death pathways.

**Figure 14:**
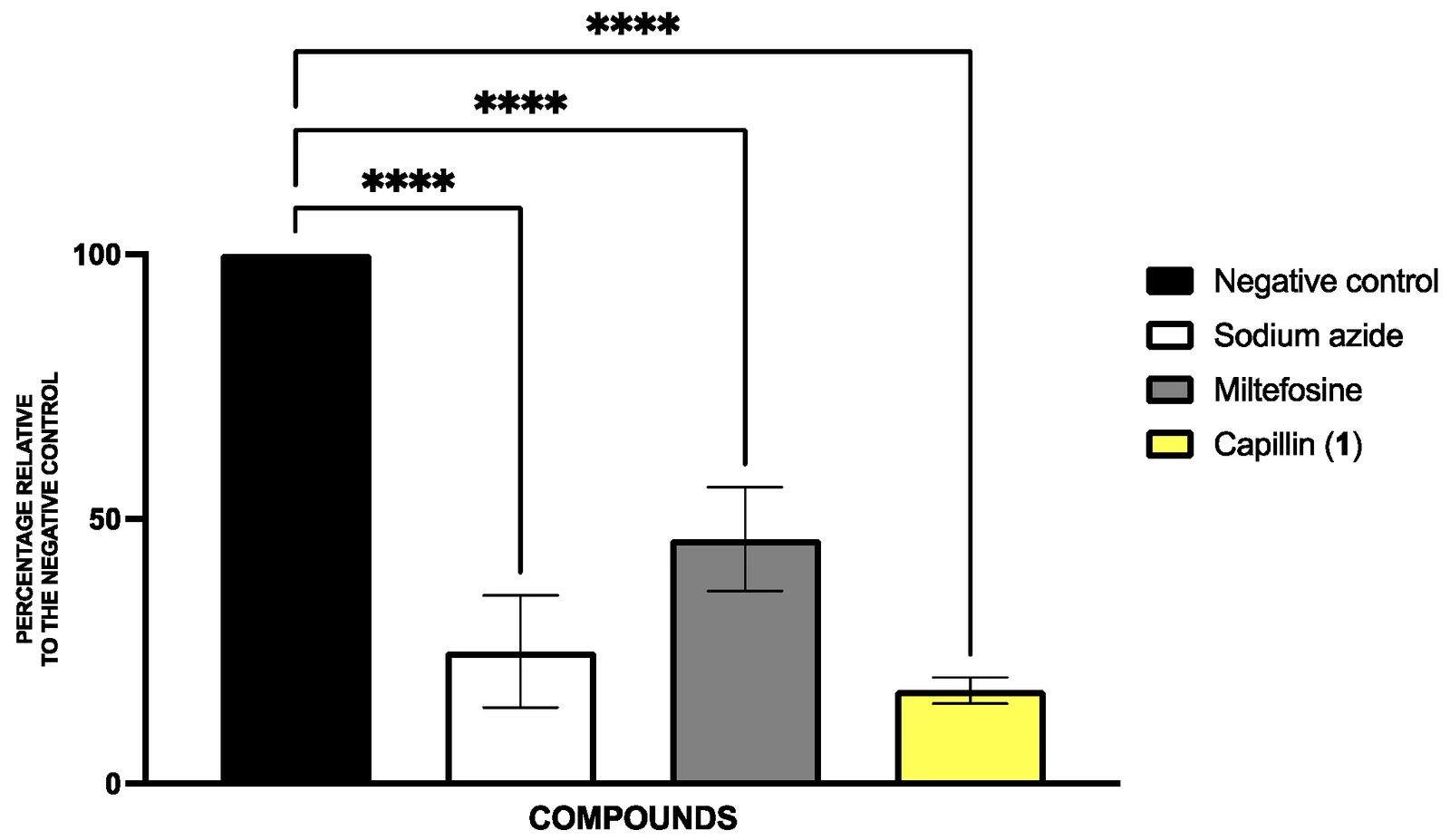
Quantification of the ATP levels with the CellTiter-Glo Luminescent Cell Viability Assay kit in *L. amazonensis*. The luminescence was measured using an EnSpire Multimode Plate Reader^®^. Miltefosine was used as the reference treatment and 20 mM sodium azide as the positive control. For each treatment, parasites were quantified from three independent replicates, and a Tukey test was performed using GraphPad PRISM^®^ 10.1.1 software to assess statistical differences between means relative to the negative control. Statistical significance was set at *p <* 0.0001 (****).

**Figure 15:**
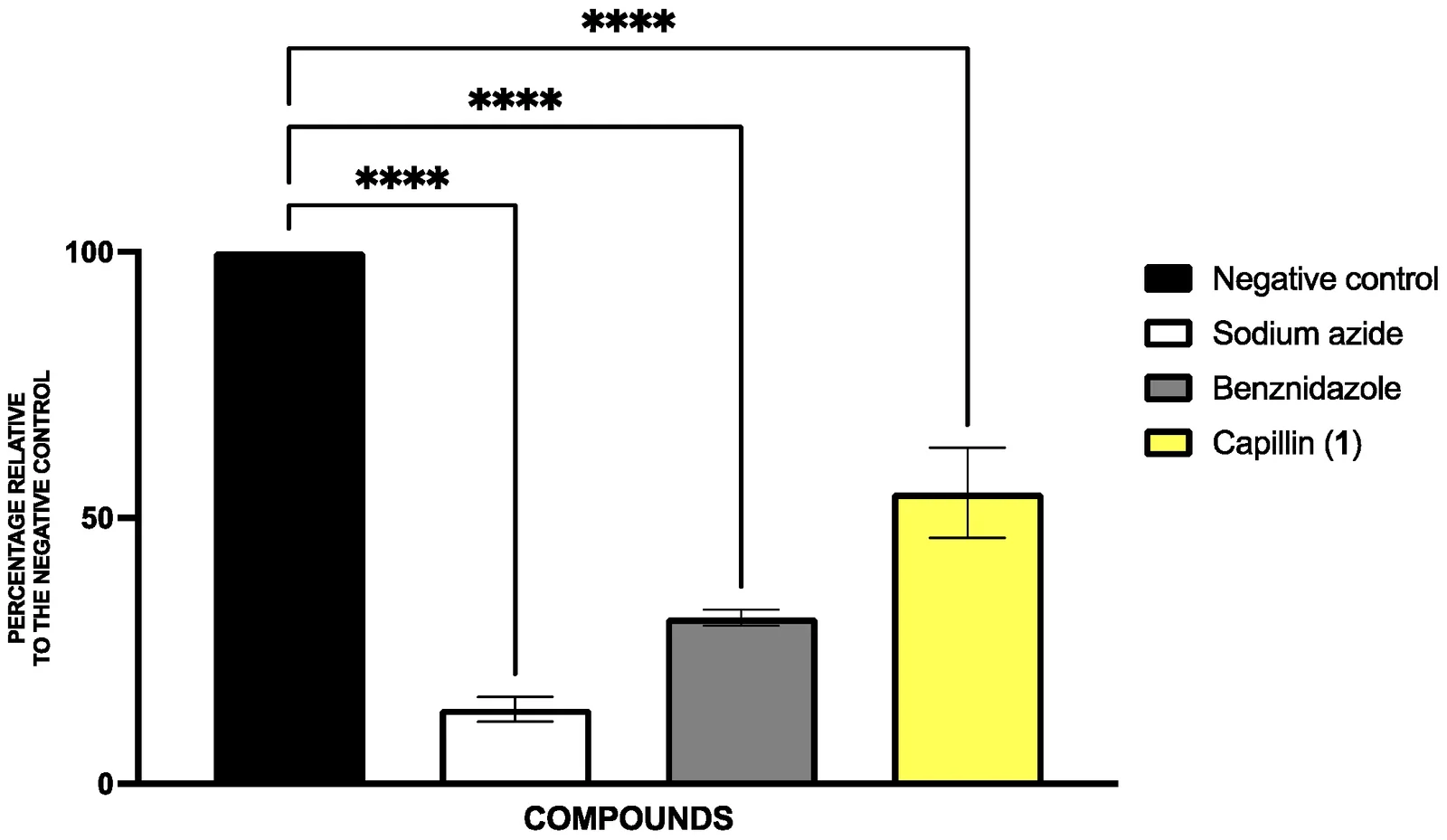
ATP level quantification with the CellTiter-Glo Luminescent Cell Viability Assay kit in *T. cruzi*. The luminescence was measured using an EnSpire Multimode Plate Reader^®^. Benznidazole was used as the reference treatment and 20 mM sodium azide as the positive control. For each treatment, parasites were quantified from three independent replicates, and a Tukey test was performed using GraphPad PRISM^®^ 10.1.1 software to assess statistical differences between means relative to the negative control. Statistical significance was set at *p* < 0.0001 (****).

Alteration of the mitochondrial membrane potential (ΔΨm) and impaired ATP production promote apoptosis through the mitochondrial pathway. To measure alterations in ATP levels, the CellTiter-Glo^®^ Luminescent Cell Viability Assay Kit was used. The ATP levels of *L. amazonensis* (Figure 14) and *T. cruzi* (Figure 15) were expressed as a percentage of the negative control. Sodium azide was used as the positive control, and the results were compared with those of the reference drugs miltefosine and benznidazole, which are used to treat leishmaniasis and Chagas disease, respectively. Capillin reduced ATP production by 75% in *L. amazonensis* and by 50% in *T. cruzi* compared to the negative control.

**Figure 16:**
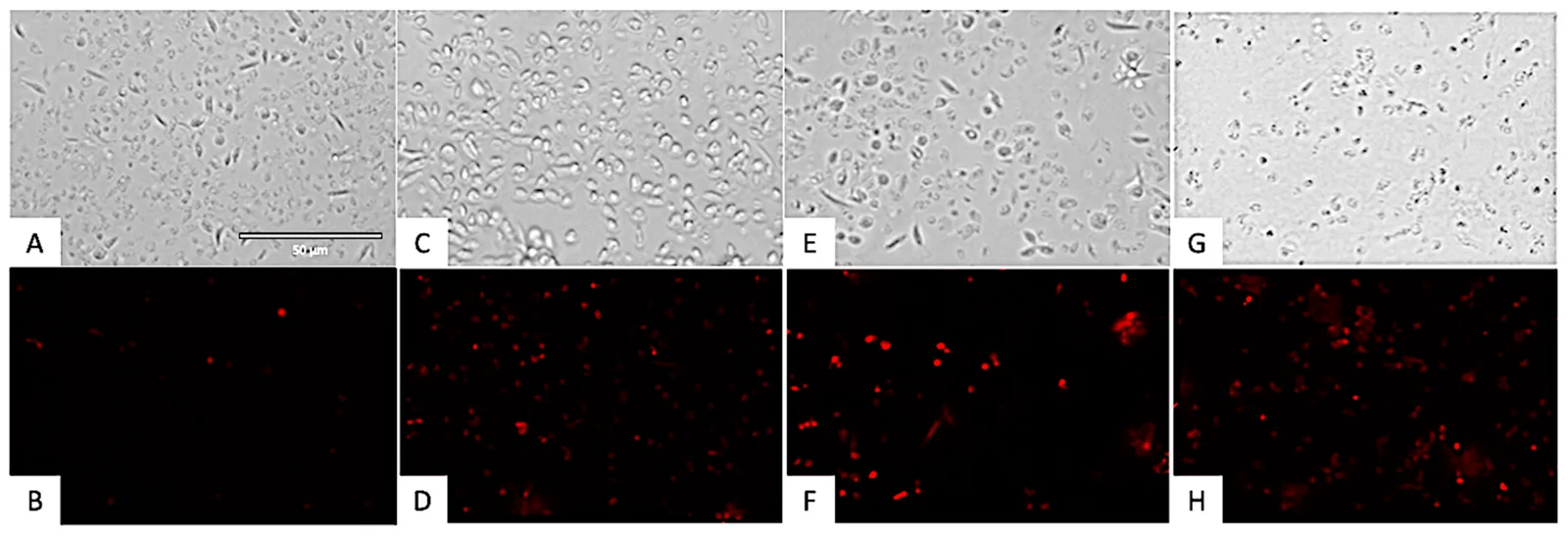
Representative images of the Cell ROX^®^ Deep Red fluorescent probe in *L. amazonensis*. The images were taken using the EVOS M5000 Imaging System (40×) after 24 h of incubation at 26 °C. (**A**) Untreated parasites in the visible channel. (**B**) Untreated parasites in the Cy5 channel. (**C**) Parasites treated with 600 µM H_2_O_2_ in the visible channel. (**D**) Parasites treated with 600 µM H_2_O_2_ in the Cy5 channel. (**E**) Parasites treated with the IC_90_ of miltefosine in the visible channel. (**F**) Parasites treated with the IC_90_ of miltefosine in the Cy5 channel. (**G**) Parasites treated with the IC_90_ of capillin in the visible channel. (**H**) Parasites treated with the IC_90_ of capillin in the Cy5 channel. Scale bar: 50 µm.

**Figure 17:**
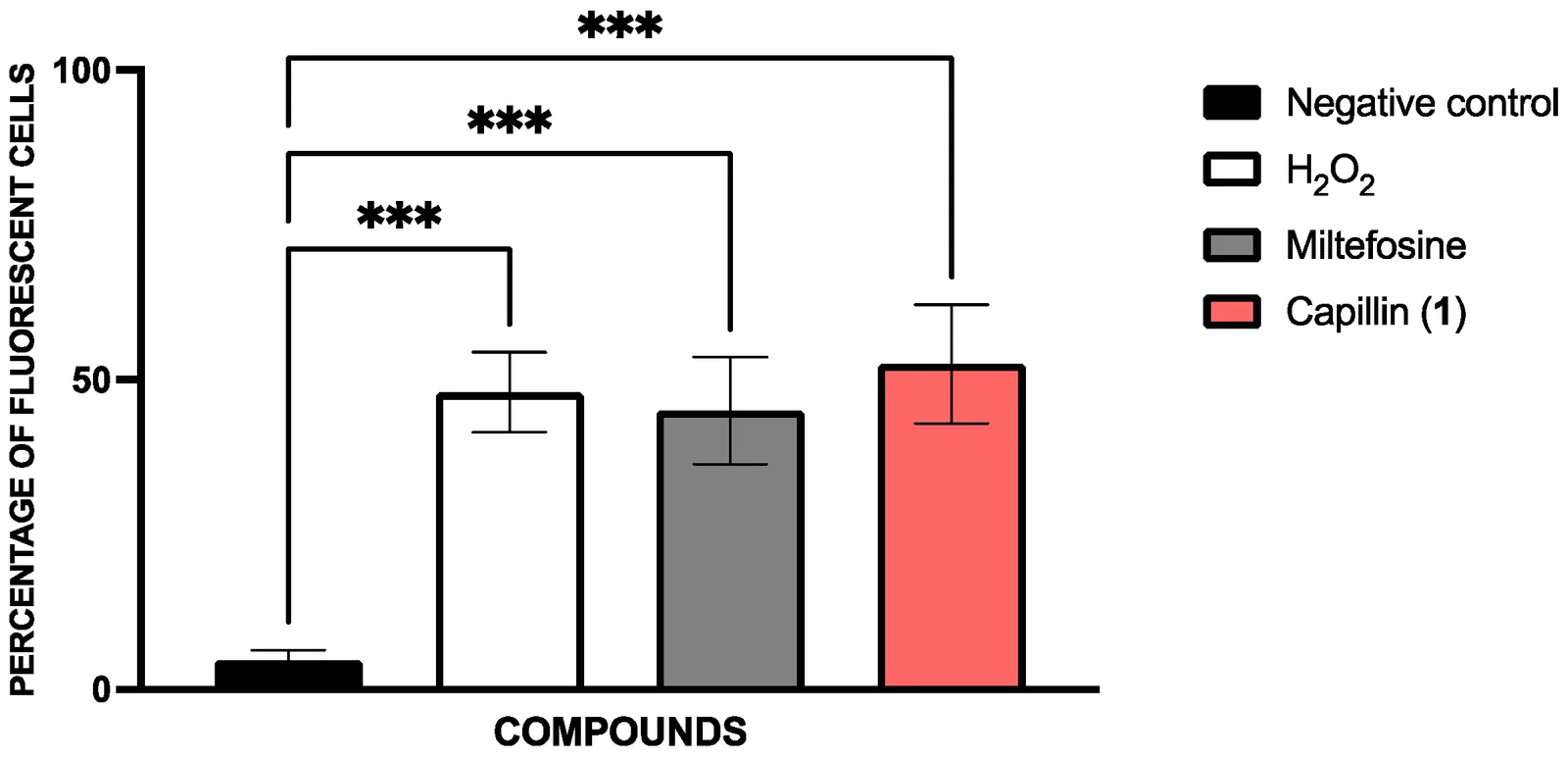
ROS level quantification with the Cell ROX^®^ Deep Red fluorescent probe assay in *L. amazonensis*. The parasites were counted manually using the EVOS M5000 Imaging System software. Miltefosine was used as the reference treatment and 600 µM hydrogen peroxide (H_2_O_2_) as the positive control. For each treatment, parasites were quantified from three independent replicates, with at least 200 cells counted in each image, and a Tukey test was performed using GraphPad PRISM^®^ 10.1.1 software to assess statistical differences between means relative to the negative control. Statistical significance was set at *p* < 0.001 (***).

**Figure 18:**
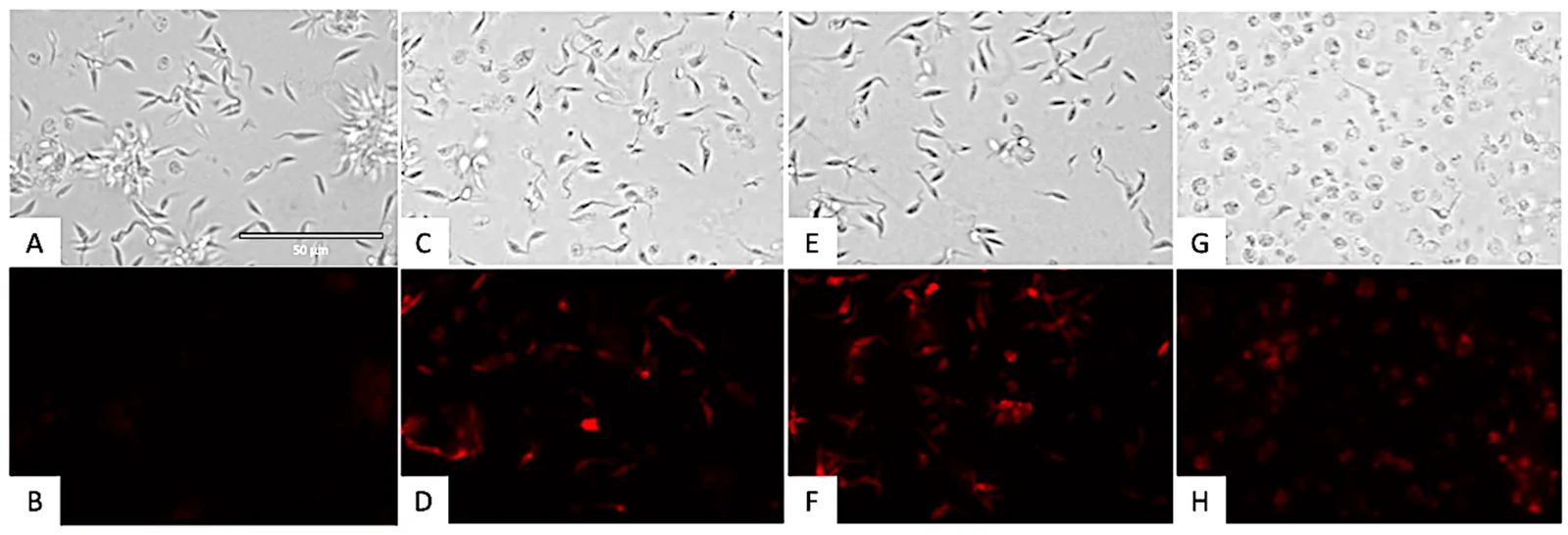
Representative images of the Cell ROX^®^ Deep Red fluorescent probe in *T. cruzi*. The images were taken using the EVOS M5000 Imaging System (40×) after 24 hof incubation at 26 °C. (**A**) Untreated parasites in the visible channel. (**B**) Untreated parasites in the Cy5 channel. (**C**) Parasites treated with 600 µM H_2_O_2_ in the visible channel. (**D**) Parasites treated with 600 µM H_2_O_2_ in the Cy5 channel. (**E**) Parasites treated with the IC_90_ of benznidazole in the visible channel. (**F**) Parasites treated with the IC_90_ of benznidazole in the Cy5 channel. (**G**) Parasites treated with the IC_90_ of capillin in the visible channel. (**H**) Parasites treated with the IC_90_ of capillin in the Cy5 channel. Scale bar: 50 µm.

**Figure 19:**
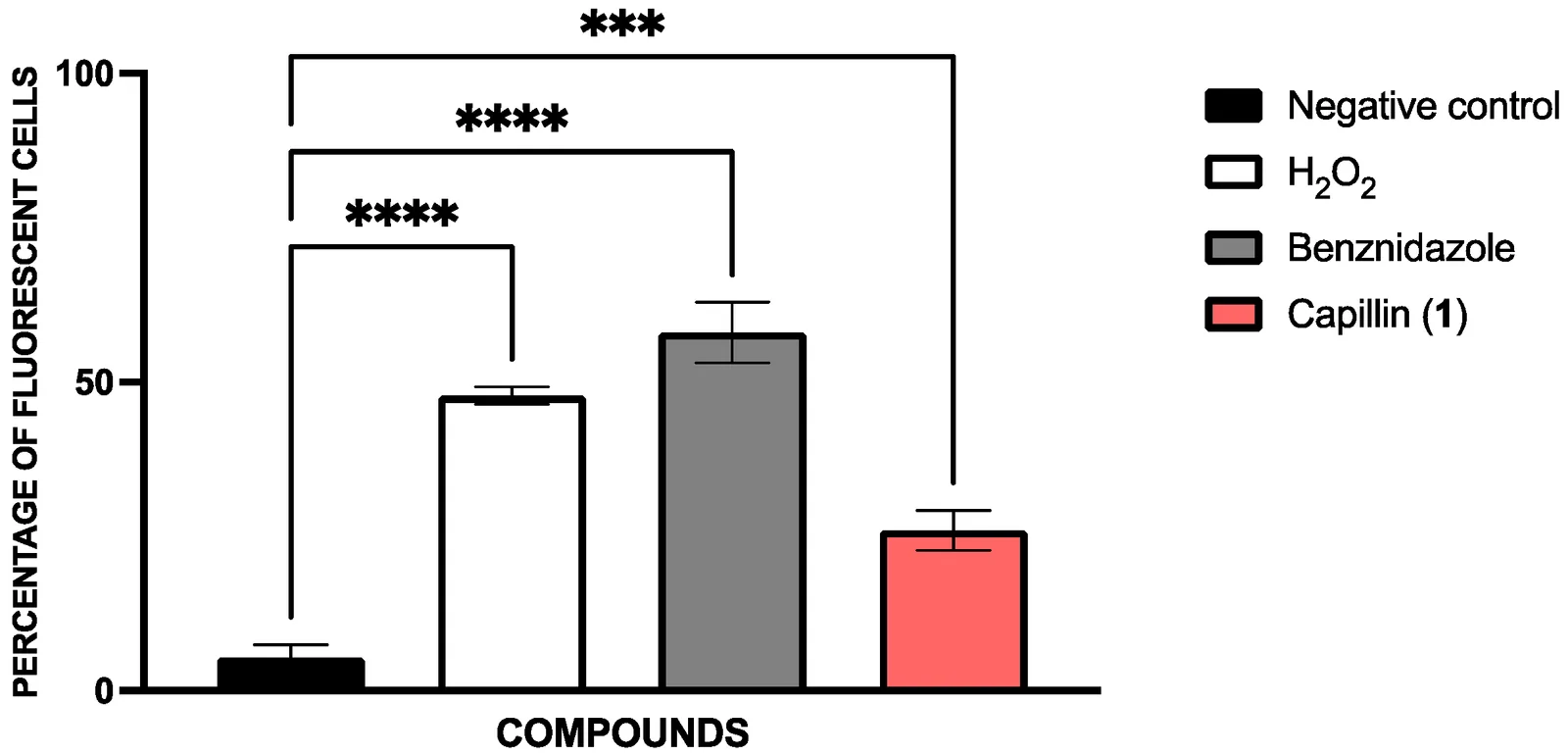
ROS level quantification with the Cell ROX^®^ Deep Red fluorescent probe assay in *T. cruzi*. The parasites were counted manually using the EVOS M5000 Imaging System software. Benznidazole was used as the reference treatment and 600 µM hydrogen peroxide (H_2_O_2_) as the positive control. For each treatment, parasites were quantified from three independent replicates, with at least 200 cells counted in each image, and a Tukey test was performed using GraphPad PRISM^®^ 10.1.1 software to assess statistical differences between means relative to the negative control. Statistical significance was set at *p* < 0.001 (***) and *p* < 0.0001 (****).

Another hallmark physiological alteration that triggers programmed cell death (PCD) in kinetoplastid protozoa is the intracellular accumulation of reactive oxygen species (ROS). Mitochondrial dysfunction perturbs redox homeostasis, causing an imbalance between free radicals and antioxidants [45,46]. To assess the oxidative stress induced by capillin, intracellular ROS accumulation was measured using the Cell ROX™ Deep Red Flow Cytometry Assay Kit in *L. amazonensis* (Figure 16 and Figure 17) and *T. cruzi* (Figure 18 and Figure 19). Capillin treatment resulted in markedly elevated red fluorescence, consistent with oxidative stress induction in both parasites.

Our findings align with published data reporting that capillin [34], isolated from the essential oil of the flowers of *Artemisia capillaris* Thunb., induces apoptosis in human HL-60 leukemia cells through the mitochondrial pathway.

Overall, the mechanistic results support the notion that compound **1** induces programmed cell death-like events in *T. cruzi* and *L. amazonensis*. These findings indicate that apoptosis is mediated by mitochondrial dysfunction, accompanied by increased membrane permeability and chromatin condensation.

## 4. Conclusions

Chagas disease and leishmaniasis continue to represent significant public health challenges, highlighting the urgent need for the development of new therapeutic agents. Through bioassay-guided fractionation using leishmanicidal and trypanocidal assays, the ethanolic root extract of cultivated *A. frutescens* yielded several polyacetylenes. Among these, capillin (1) exhibited strong antikinetoplastid potency comparable to or exceeding that of the standard therapeutic agents miltefosine and benznidazole. It also displayed a high selectivity index in a mammalian cell line. Preliminary SAR analyses suggested that the type of functional group plays a key role in the antiparasitic activity. Additionally, compound **1** triggers events consistent with programmed cell death in *T. cruzi* and *L. amazonensis*. Furthermore, the isolated compounds could serve as a basis for structural optimization and the development of more effective leishmanicidal and trypanocidal agents. Overall, these findings highlight *A. frutescens* as a valuable source of bioactive polyacetylenes with leishmanicidal and trypanocidal potential. These compounds represent promising candidates for further investigation as potential therapeutic agents, supporting the continued exploration of this species in the search for alternatives or complements to current antiparasitic drugs.

## Figures and Tables

**Table 1:** Trypanocidal activity of isolated polyacetylenes **1–4** against the epimastigote stage of *T. cruzi* and cytotoxicity against murine macrophage cell line J774A.1.

Compounds	*T. cruzi*		Cytotoxicity
	IC_50_^a^	SI ^b^	CC_50_^c^
**1**	0.11 ± 0.01	1815.5	199.70 ± 3.37
**2**	2.70 ± 0.04	72.2	194.90 ± 6.12
**3**	4.10 ± 0.10	38.0	155.80 ± 7.07
**4**	2.50 ± 0.14	2.9	7.30 ± 1.79
**B** ^d^	6.94 ± 1.90	57.6	399.91 ± 1.40

*^a^* IC_50_: Concentration able to inhibit 50% of parasites expressed as µM ± standard deviation (SD). *^b^* SI: selectivity index (CC_50_/IC_50_). *^c^* CC_50_: Concentration able to inhibit 50% of murine macrophages expressed as µM ± standard deviation (SD). *^d^* B: Benznidazole was used as the positive control. All assays were performed as independent experiments on three different days.

**Table 2:** Leishmanicidal activity of isolated polyacetylenes **1**–**4** against the promastigote stage of *Leishmania* spp. and cytotoxicity against murine macrophage cell line J774A.1.

Compounds	*L. amazonensis*		*L. donovani*		Cytotoxicity
	IC_50_^a^	SI ^b^	IC_50_ ^a^	SI ^b^	CC_50_^c^
**1**	0.70 ± 0.11	285.3	6.50 ± 1.41	30.7	199.70 ± 3.37
**2**	8.10 ± 0.60	24.1	42.20 ± 7.90	4.6	194.90 ± 6.12
**3**	37.60 ± 1.90	4.1	129.30 ± 16.40	1.2	155.80 ± 7.07
**4**	3.80 ± 0.20	1.9	26.04 ± 8.20	0.3	7.30 ± 1.79
**M** ^d^	6.48 ± 0.25	11.1	3.32 ± 0.04	21.7	72.18 ± 8.85

*^a^* IC_50_: Concentration able to inhibit 50% of parasites expressed as µM ± standard deviation (SD). *^b^* SI: selectivity index (CC_50_/IC_50_). *^c^* CC_50_: Concentration able to inhibit 50% of murine macrophages expressed as µM ± standard deviation (SD). *^d^* M: Miltefosine was used as the positive control. All assays were performed as independent experiments in triplicate.

**Table 3:** Leishmanicidal and trypanocidal activity of polyacetylene **1** against the intracellular forms of *L. amazonensis* and *T. cruzi*, and cytotoxicity against murine macrophage cell line J774A.1.

Compounds	*L. amazonensis*		*T. cruzi*		Cytotoxicity
	IC_50_^a^	SI ^b^	IC_50_^a^	SI ^b^	CC_50_ ^c^
**1**	1.70 ± 0.08	117.5	5.30 ± 0.20	37.7	199.7 ± 3.37
**M** ^d^	3.12 ± 0.30	23.1			72.18 ± 8.85
**B** ^e^			2.67 ± 0.39	149.8	399.91 ± 1.40

*^a^* IC_50_: Concentration able to inhibit 50% of parasites expressed as µM ± standard deviation (SD). *^b^* SI: selectivity index (CC_50_/IC_50_). *^c^* CC_50_: Concentration able to inhibit 50% of murine macrophages expressed as µM ± standard deviation (SD). ^*d*^ M: Miltefosine was used as the positive control against *L. amazonensis*. *^e^* B: Benznidazole was used as the positive control against *T. cruzi*. All assays were performed as independent experiments in triplicate.

## Data Availability

The data supporting the findings of this study are available within the article.
